# Quantifying hexafluoroisopropanol's hydrogen bond donor ability: infrared photodissociation spectroscopy of halide anion HFIP complexes†

**DOI:** 10.1039/d4sc08456j

**Published:** 2025-01-29

**Authors:** Milena Barp, Florian Kreuter, Qian-Rui Huang, Jiaye Jin, Franka. E. Ninov, Jer-Lai Kuo, Ralf Tonner-Zech, Knut R. Asmis

**Affiliations:** a Wilhelm-Ostwald-Institut für Physikalische und Theoretische Chemie, Universität Leipzig Linnéstraße 2 04103 Leipzig Germany ralf.tonner@uni-leipzig.de knut.asmis@uni-leipzig.de; b Institute of Atomic and Molecular Sciences Academia Sinica No. 1 Roosevelt Rd, Sec 4 Taipei 106319 Taiwan jlkuo@gate.sinica.edu.tw

## Abstract

We report on the gas phase vibrational spectroscopy (3500–950 cm^−1^) of halide anion complexes with 1,1,1,3,3,3-hexafluoroisopropanol (HFIP) and its partially deuterated analogue (HFIP-*d*_1_). Infrared photodissociation spectra of messenger-tagged X^−^(HFIP/HFIP-*d*_1_), with X^−^ = Cl^−^, Br^−^, and I^−^, together with electronic structure calculations reveal O–H(D) stretching fundamentals that are red-shifted twice as much as those for the corresponding complexes with isopropanol and water, directly reflecting HFIP's enhanced hydrogen-bond donor ability. The harmonic analysis of the bands in the fingerprint region reveals that HFIP assumes a synperiplanar conformation in the complexes. The consideration of anharmonic effects is necessary to recover the efficient coupling between stretching and bending modes in the OH stretching region. An energy decomposition analysis shows that the roughly twice as large binding energy in the HFIP complexes *vs. i*-PrOH and water is determined mainly by differences in the electrostatic attraction. The observed red-shifts, which reflect the extent of charge transfer along the coordinate of the proton transfer reaction X^−^ + HM → XH + M^−^, correlate qualitatively with the difference in the proton affinities ΔPA = PA(X^−^) − PA(M^−^). A more quantitative agreement requires also considering differences in the hydrogen bond angle.

## Abstract 2

We quantifying hexafluoroisopropanol's hydrogen bond (HB) donor ability by ion vibrational spectroscopy. The observed red-shifts reflect the extent of charge transfer along the proton transfer coordinate and correlate with the difference in the proton affinities of the two HB acceptors.

## Graphical abstract

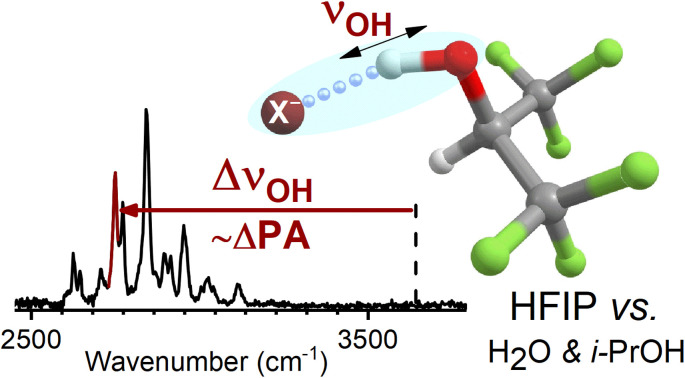

## 1 Introduction

1,1,1,3,3,3-Hexafluoroisopropanol (HFIP) is a versatile, cost-effective and increasingly popular solvent used in organic synthesis for promoting chemical reactions,^1,2^ like epoxidation,^3^ C–H bond activation,^4^ regioselective halogenation,^5^ biomimetic polyene cyclization,^6^ electrochemical oxidative cross-coupling,^7^ and late-stage deuterations of aromatic compounds.^8^ HFIP also finds application in chemical biology (*e.g.* protein structure determination) as well as supramolecular and polymer science.^1^ Its catalytic properties are mainly traced back to its high polarity, increased Brønsted acidity, high ionizing power, and low nucleophilicity combined with a strong hydrogen-bond (HB) donation and network formation ability.^1,2,9^ While for some reactions, aided by electronic structure calculations, a molecular-level reaction mechanism has been proposed, see for instance ref. 3, 8 and 10 for particularly intriguing examples, for many of the more recent studies the role of HFIP remains unknown.^1^ In order to gain a better understanding of HFIP's properties in solution, a reliable description of its intermolecular interactions is important. In this regard, useful insights can be obtained from experimental studies on gas-phase species, which allow isolating such interactions. Such model systems are also amenable to higher level quantum chemical calculations that can then be used to benchmark lower level methods applicable to extended condensed phase systems.

**Scheme 1:**
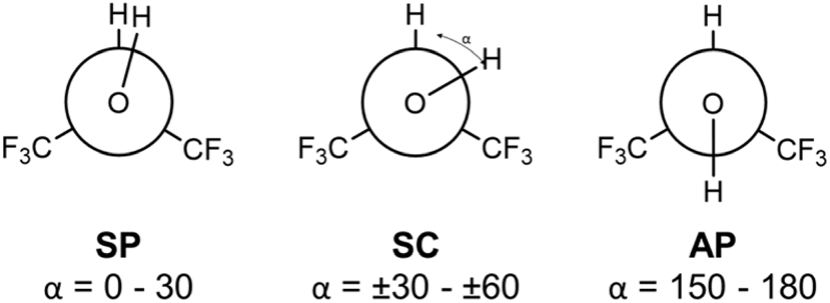


Infrared (IR) studies show that the isolated HFIP molecule exists in two conformations, an antiperiplanar (AP) and a synclinal (SC) conformer (see Scheme 1).^11^ The AP conformer is found to be more stable, in contrast to HFIP's non-fluorinated analog isopropanol (*i*-PrOH), which adopts an SC conformation.^12^ Shahi and Arunan reported the microwave spectrum of HFIP cooled in a supersonic expansion and assigned it to the AP conformer.^13^ Their *ab initio* calculations confirm this assignment and find the SC conformer about 5 kJ mol^−1^ higher in energy, while the synperiplanar (SP) conformer represents a saddle point on the potential energy surface, only 1 kJ mol^−1^ above the SC minima.^13^ HFIP's dipole moment increases along the series AP → SC → SP.^14,15^ As a result, the higher energy structures are stabilized upon aggregation as a consequence of more favorable electrostatic interactions, leading to the stronger HB donor ability of aggregated HFIP.^3,14^

Wang and coworkers recently studied orientation-specific charge–dipole interactions in the anion complexes X^−^(HFIP), X^−^ = F^−^, Cl^−^, Br^−^, I^−^, and O_2_^−^, using anion photoelectron spectroscopy.^16,17^ They showed that the combination of the charge–dipole interaction with the formation of an ionic hydrogen bond (IHB) leads to a preference for the SP/SC isomer, since these HFIP conformers exhibit a larger dipole moment. The interaction energy decreases with increasing anion size, *i.e.*, with decreasing anion proton affinity (PA). The interaction with the fluoride anion is considerably stronger than with the chloride anion, leading to proton transfer and formation of a complex formally containing HF and deprotonated HFIP.^16^ This directly raises the question regarding the role of charge transfer (*vs.* electrostatic interactions) in these model systems containing IHBs.^18,19^

Here, we apply cryogenic ion trap vibrational spectroscopy^20^ to study the halide anion complexes X^−^(HM) with X^−^ = Cl^−^, Br^−^, I^−^ and HM = HFIP, HFIP-*d*_1_, *i*-PrOH, *i*-PrOD, H_2_O and D_2_O. The present study builds on the landmark studies of Johnson and coworkers, who studied the IHB interaction in halide anion complexes with water in the gas phase.^21,22^ They demonstrated that vibrational action spectroscopy allows to systematically characterize IHBs by measuring the red-shift of the corresponding OH stretching frequency (Δ*ν*_OH_), which is defined as

1 Δ*ν*_OH_ = *ν*^free^_OH_ − *ν*^HB^_OH_,

where *ν*^HB^_OH_ and *ν*^free^_OH_ correspond to the fundamental stretching vibrational frequencies (in cm^−1^) of the hydrogen-bonded and of the corresponding free, uncoupled OH oscillator, respectively. Δ*ν*_OH_ correlates with the HB strength.^23^ Combined with the results from *ab initio* calculations, we assign the obtained vibrational spectra to a particular isomer. While the bands in the fingerprint region can be assigned based on a harmonic analysis, the consideration of anharmonic effects is necessary to disentangle the pronounced coupling between stretching and bending modes in the OH stretching region. Based on an energy decomposition analysis we dissect the interactions that contribute to HFIP's HB donor ability, compare these results to those obtained for the related halide anion complexes with isopropanol and water and then discuss the role of electrostatic interactions *vs.* charge transfer in the larger context of proton transfer reactions.

## 2 Results

We start by presenting the IRPD spectra of the messenger-tagged halide anion complexes X^−^(HFIP) with X^−^ = Cl^−^, Br^−^, and I^−^. The spectral signature observed in the OH stretching region (3500–1950 cm^−1^) yields characteristic values for Δ*ν*_OH_, which are directly related to the HB strength and hence allow quantifying HFIP's HB donor ability. To compare these red-shifts to those of other common solvent molecules, we also measured the IRPD spectra of the corresponding messenger-tagged complexes X^−^(*i*-PrOH) and X^−^(H_2_O). The IRPD spectra obtained in the fingerprint region (1525–950 cm^−1^) can readily be assigned based on a harmonic frequency analysis and allow identifying the particular stereoisomer present in the experiments. We also consider the deuterated isotopologues X^−^(HFIP-*d*_1_), X^−^(*i*-PrOD) and X^−^(D_2_O) in our study to evaluate the role of vibrational anharmonicities in IHBs (see Analysis section).

### 2.1 X^−^(HFIP)

**Fig. 1:**
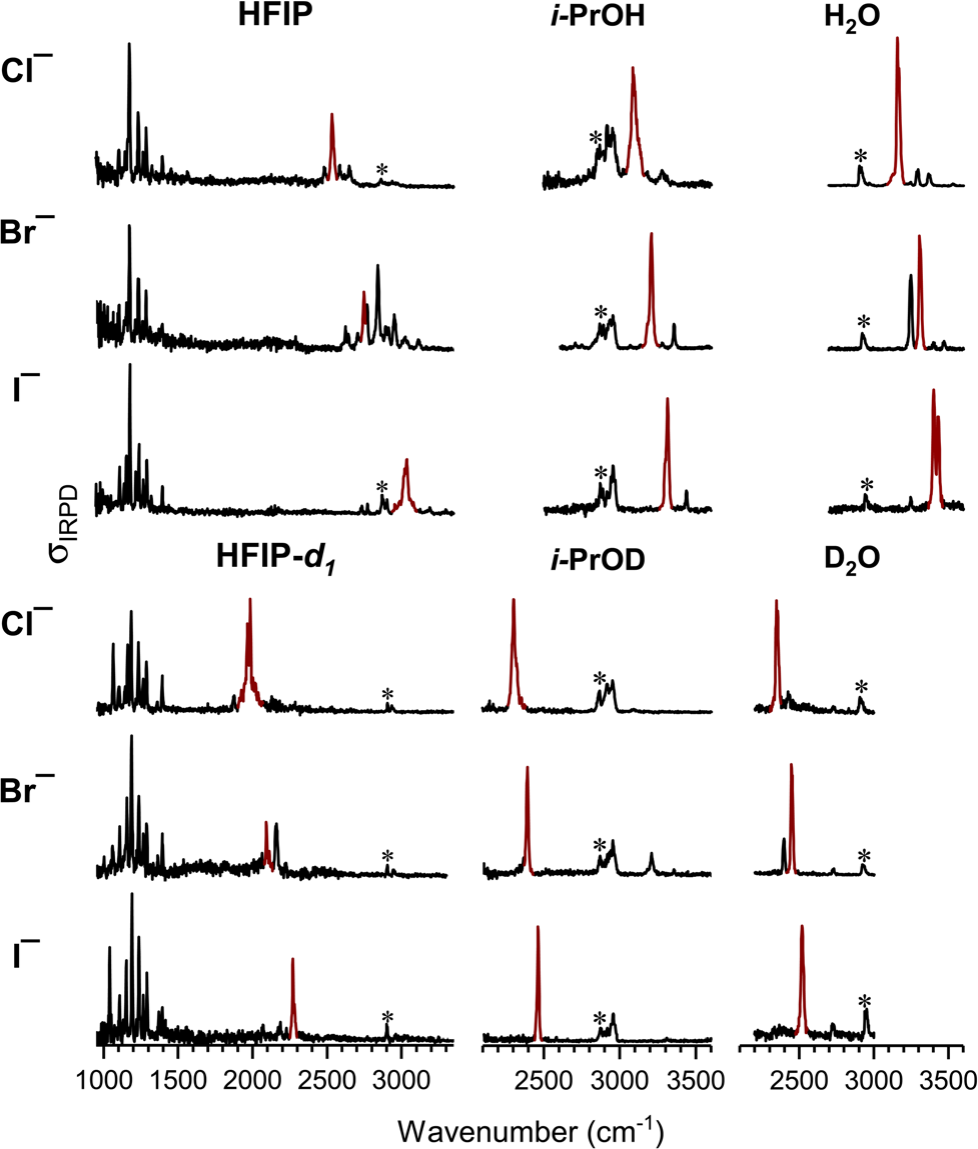
IRPD spectra of D_2_-tagged X^−^(HFIP), X^−^(*i*-PrOH) and X^−^(H_2_O) (upper panels, from left to right) and X^−^(HFIP-*d*_1_), X^−^(*i*-PrOD) and X^−^(D_2_O) complexes (lower panels, from left to right) for X^−^ = Cl^−^, Br^−^ and I^−^ (top to bottom). Bands assigned to the OH(OD) stretching fundamental are shown in red (see Table 1 for band positions). Bands marked with an asterisk indicate excitation of the D_2_ stretching mode of the messenger-tag.

IRPD spectra of D_2_-tagged Cl^−^(HFIP), Br^−^(HFIP), I^−^(HFIP), Cl^−^(HFIP-*d*_1_), Br^−^(HFIP-*d*_1_) and I^−^(HFIP-*d*_1_) (from top to bottom), measured in the spectral region from 3350 cm^−1^ down to 950 cm^−1^, are shown in the left part of Fig. 1. First, we focus our attention on the general trends observed in the OH and OD stretching region. Experimental and calculated stretching frequencies *ν*_OH_ (*ν*_OD_) and red-shifts Δ*ν*_OH_ (Δ*ν*_OD_) are listed in Table 1.

**Table 1:** Experimental band position (in cm^−1^) of the OH(OD) stretch fundamentals (*ν*_OH_, *ν*_OD_) and OH(OD) red-shifts (Δ*ν*_OH_, Δ*ν*_OD_) obtained from the IRPD spectra of D_2_-tagged X^−^(HM) complexes (HM = HFIP/HFIP-*d*_1_, *i*-PrOH/*i*-PrOD, H_2_O/D_2_O) shown in Fig. 1 compared to the corresponding harmonic and anharmonic vibrational frequencies (in cm^−1^) of the untagged complexes^f^

Complex	*ν*_OH_/*ν*_OD_				Δ*ν*_OH_/Δ*ν*_OD_
	IRPD^a^	Harmonic^b^	VPT2^c^	DVR-FBR^d^	IRPD^e^
Cl^−^(HFIP/HFIP-*d*_1_)	2535/1973	2925/2134	2441/1880	2588/1993	1133/730
Br^−^(HFIP/HFIP-*d*_1_)	2748/2092	3091/2251	2719/2079	2731/2080	920/611
I^−^(HFIP/HFIP-*d*_1_)	3028/2271	3219/2344	2928/2204		640/432
Cl^−^(*i*-PrOH/*i*-PrOD)	3087/2303	3270/2383	2994/2246		571/373
Br^−^(*i*-PrOH/*i*-PrOD)	3208/2393	3365/2450	3136/2340		450/283
I^−^(*i*-PrOH/*i*-PrOD)	3315/2463	3460/2519	3230/2403		343/213
Cl^−^(H_2_O/D_2_O)	3158/2350	3338/2425	3081/2294		549/380
Br^−^(H_2_O/D_2_O)	3308/2449	3426/2487	3223/2391		399/281
I^−^(H_2_O/D_2_O)	3417/2520	3525/2557	3328/2461		290/210

a Values obtained from IRPD spectra of D_2_-tagged complexes. The value reported is for the most intense IRPD band assigned to the OH/OD stretch fundamental (see text for details). For I^−^(H_2_O) and Cl^−^(HFIP-*d*_1_) we report the center of the doublet, see ref. 22 and 24 for detailed band assignment of I^−^(H_2_O).

b MP2/aug-cc-pVTZ (X^−^ = Cl^−^, Br^−^) or MP2/aug-cc-pVTZ-PP (X^−^ = I^−^) harmonic frequencies.

c VPT2/MP2/aug-cc-pVDZ (X^−^ = Cl^−^, Br^−^) or VPT2/MP2/aug-cc-pVDZ-PP (X^−^ = I^−^) anharmonic frequencies.

d DVR-FBR/RI-MP2 + DLPNO-CCSD(T)/aug-cc-pVTZ anharmonic frequencies. The value reported is for the transition with highest OH stretch contribution (see text for details).

e Red-shifts are determined with respect to the vibrational frequency of the corresponding free, uncoupled OH or OD oscillator: HFIP (SC, 3668 cm^−1^),^11,25^ H_2_O (3707 cm^−1^),^26,27^*i*-PrOH (3658 cm^−1^),^12^ HFIP-*d*_1_ (SC 2703 cm^−1^),^25^ D_2_O (2730 cm^−1^),^28^ and *i*-PrOD (2676 cm^−1^).^29^

f See Methods section and ESI for computational details and references.

Above 1800 cm^−1^, excitation of the OH stretching mode (*ν*_OH_) of the hydroxyl group involved in the IHB is the most prominent and also the most diagnostic feature. Note, multiple bands associated with this excitation are observed in several spectra. Similar observations have been previously reported for halide–water complexes and attributed to the presence of a strong IHB combined with the anharmonic nature of the corresponding O–H oscillator, leading to the excitation of combination bands.^21,24^ Moreover, Fermi resonances with nearby overtone excitations of the CH and OH bending modes can further complicate the spectral pattern.^24^ The most intense band (above 1800 cm^−1^) typically corresponds to the fundamental excitation of the OH stretching mode, except for the spectra of Br^−^(HFIP) and Br^−^(HFIP-*d*_1_), where strong anharmonic coupling leads to two bands of similar intensity (*vide infra*). For Cl^−^(HFIP), Br^−^(HFIP) and I^−^(HFIP), the bands assigned to excitation of the OH stretching fundamental are centered at 2535 cm^−1^, 2748 cm^−1^ and 3028 cm^−1^, respectively, corresponding to decreasing red-shifts Δ*ν*_OH_ of 1133 cm^−1^ (Cl^−^), 920 cm^−1^ (Br^−^) and 640 cm^−1^ (I^−^) with increasing halide anion size, as expected. Δ*ν*_OH_ is determined with respect to the OH stretching frequency of the free SC conformer of HFIP (3668 cm^−1^)^25^ and is also listed in Table 1. There are two substantially weaker features that we also observed in this spectral region, which correspond to excitation of the CH stretching mode of HFIP (2935–2958 cm^−1^) as well as the nominally IR-forbidden stretching mode of D_2_ (2862–2905 cm^−1^), which gains IR intensity through charge-induced-dipole interactions.^30^

Upon deuteration of HFIP's hydroxyl group the corresponding IRPD feature, now associated with the OD stretch excitation (Δ*ν*_OD_), is shifted to lower wavenumbers by a factor of 1.29–1.33, close to the expected ratio of 1.36 for a free OH *vs.* a free OD oscillator. For chloride, bromide and iodide, Δ*ν*_OD_ is observed at 1973 cm^−1^ (Δ*ν*_OD_ = 730 cm^−1^), 2092 cm^−1^ (611 cm^−1^) and 2271 cm^−1^ (432 cm^−1^), respectively. Moreover, the associated absorption features are simpler, indicating that anharmonic couplings are reduced upon deuteration, as expected.

### 2.2 X^−^(*i*-PrOH)

In order to evaluate the observed red-shifts Δ*ν*_OH_ and Δ*ν*_OD_ for the HFIP-containing complexes, we compare them to those of the corresponding anion–molecule complexes containing *i*-PrOH and its partially deuterated isotopologue *i*-PrOD. The IRPD spectra of the D_2_-tagged complexes (>2200 cm^−1^) are shown in the center column of Fig. 1 and the OH (OD) stretching frequencies are listed in Table 1.

Similar to the previously discussed IRPD spectra, the most intense transition in this spectral region is due to excitation of the hydrogen-bonded hydroxyl group and observed at 3087 cm^−1^ (Cl^−^), 3208 cm^−1^ (Br^−^) and 3315 cm^−1^ (I^−^) for the three halide complexes. These correspond to red-shifts that are roughly half as large as for the corresponding HFIP complexes. Due to the weaker HBs, the OH stretching features are simpler than those observed for HFIP. Nonetheless, there is some unresolved structure observed in the most intense IRPD feature, indicating efficient coupling to a low frequency mode, presumably to the HB stretching mode. There are also one or two bands at higher wavenumbers, which we attribute to the excitation of combination bands. At lower energies, around 3000 cm^−1^ and below, excitation of the seven CH stretching modes contributes to a partially resolved feature consisting of multiple vibrational transitions. The D_2_ stretch of the tagging molecule is also expected in this region (see Fig. 1).

Upon deuteration of *i*-PrOH's hydroxyl group, an OD stretching band is observed below 2500 cm^−1^ at 2303 cm^−1^ (Cl^−^), 2393 cm^−1^ (Br^−^) and 2463 cm^−1^ (I^−^), corresponding to *ν*_OH_/*ν*_OD_ ratios of 1.34–1.35, respectively. Note, the *ν*_OD_ feature in the X^−^(*i*-PrOD)·D_2_ spectra are simpler than those observed for X^−^(HFIP-*d*_1_)·D_2_ and now consist mainly of a single band, which only remains markedly asymmetric in the Cl^−^(HFIP-*d*_1_)·D_2_ spectrum (see Fig. 1). In contrast, the CH (and D_2_) stretching bands remain nearly unchanged in position and intensity.

### 2.3 X^−^(H_2_O)

The red-shifts Δ*ν*_OH_ and Δ*ν*_OD_ found for X^−^(*i*-PrOH)/X^−^(*i*-PrOD) are quite similar to those previously reported for X^−^(H_2_O)/X^−^(D_2_O).^24^ These previous measurements were mainly based on Ar-tagged complexes. In order to ensure better comparability with the present data set, we therefore measured the IRPD spectra of the corresponding D_2_ complexes, shown in the right column of Fig. 1.

For the D_2_-tagged X^−^(H_2_O) complexes, we observe *ν*_OH_ at 3158 cm^−1^ (Cl^−^), 3308 cm^−1^ (Br^−^) and 3417 cm^−1^ (I^−^), slightly (<25 cm^−1^) less red-shifted compared to the previously reported values of 3146 cm^−1^ (Cl^−^), 3296 cm^−1^ (Br^−^) and 3393 cm^−1^ (I^−^) using Ar-tagging, suggesting that the Ar tag is slightly more perturbing than D_2_. The determined red-shifts *ν*_OH_ are up to 15% smaller to those observed for *i*-PrOH (see Table 1). Upon deuteration, this difference is reduced to below 2 cm^−1^.

Summarizing, the red-shifts Δ*ν*_OH(D)_ obtained from IRPD spectroscopy show that HFIP is a strong HB donor, roughly twice as strong compared to *i*-PrOH and H_2_O. Hence, the question arises, what is the exact nature of the IHB interaction in X^−^(HFIP) complexes? Is the IHB strength solely due to differences in electrostatic interactions, what role does charge transfer play, and are there other, not so obvious, contributions to the binding energy?

## 3 Analysis

To characterize the structure and spectroscopy of the X^−^(HFIP) complexes and compare them to those of X^−^(*i*-PrOH) and X^−^(H_2_O) in more detail we performed electronic structure calculations. We determined minimum-energy geometries and calculated harmonic IR frequencies and intensities for the two lowest energy isomers using the MP2 method. We also considered anharmonic effects using two complementary techniques, namely, the VPT2 and DVR-FBR methods (see Methods section and ESI for details†), to assign the IR-active combination and overtone transitions.

### 3.1 Energetics and structures

**Fig. 2:**
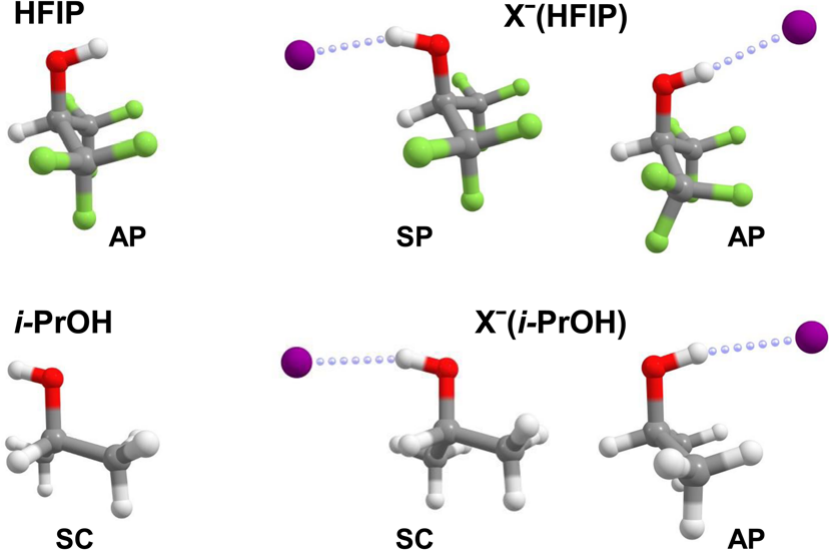
Minimum-energy structures of HFIP, *i*-PrOH, X^−^(HFIP) and X^−^(*i*-PrOH). The lowest energy conformer for the neutral molecules and the two lowest energy isomers for the anion complexes are shown. See Table 2 for relative energies and geometric parameters.

The lowest two minimum-energy structures predicted for X^−^(HFIP) complexes are compared to those for X^−^(*i*-PrOH) in Fig. 2. Their relative energies, Δ*E*, as well as characteristic geometric parameters, like the dihedral angle *φ*_HCOH_, the OH bond length *d*_OH_, the HB length *d*_HX_ and the HB angle *θ*_OHX_ are listed in Table 2 for X^−^ = Cl^−^, Br^−^, and I^−^. We also considered messenger-tag effects and find that these are negligible for evaluating the relative energies of different isomers (<1 kJ mol^−1^) as well as harmonic OH stretching frequencies (<1%) (see ESI, Fig. S14† for calculated structures and frequencies of the messenger-tagged complexes).

**Table 2:** MP2/aug-cc-pVTZ relative energies Δ*E* (in kJ mol^−1^), OH bond length *d*_OH_, HB length *d*_HX_ (both in pm), HB angle *θ*_OHX_ and dihedral angle *φ*_HCOH_ (both in degrees) for the halide anion complexes X^−^(HM) with X^−^ = Cl^−^, Br^−^, and I^−^, HM = HFIP, *i*-PrOH and H_2_O

System		Δ*E*	*d*_OH_	*d*_HX_	*θ*_OHX_	*φ*_HCOH_
Cl^−^(HFIP)	SP	0	101	192	162	0
	AP	28	103	187	175	170
Br^−^(HFIP)	SP	0	100	211	159	0
	AP	29	101	205	174	170
I^−^(HFIP)	SP	0	99	235	158	0
	AP	30	100	231	172	171
Cl^−^(*i*-PrOH)	SC	0	99	207	167	51
	AP	1.4	99	211	170	180
Br^−^(*i*-PrOH)	SC	0	99	224	165	50
	AP	1.2	99	228	169	180
I^−^(*i*-PrOH)	SC	0	98	250	163	48
	AP	0.6	98	255	170	180
Cl^−^(H_2_O)			99	212	169	
Br^−^(H_2_O)			99	229	168	
I^−^(H_2_O)			98	256	165	

The preferred conformation predicted for all X^−^(HFIP) complexes is the SP isomer (see Fig. 2), which is found at least 28 kJ mol^−1^ lower in energy than the AP isomer (see Table 2), in agreement with the results from the previous anion photoelectron spectroscopy (APES) study using DFT calculations.^16^ Interestingly, the lower energy isomer exhibits a slightly longer and hence weaker IHB than the higher energy one, independent of the nature of the halide anion. This IHB is also less linear (see Table 2), probably due to an additional, albeit, much weaker interaction between halide anion and the CH group, which is not present in the AP isomer. The driving force for formation of the SP isomer in the halide anion complexes is thus not the formation of a stronger IHB, but rather the larger dipole moment of bare HFIP's SP conformer and hence substantially larger charge–dipole interaction in the anion complex (*vide infra*).

In contrast to X^−^(HFIP), the SC isomer, the energetically favoured conformation for bare *i*-PrOH, is predicted as the global minimum-energy structure for all of the X^−^(*i*-PrOH) complexes considered here. However, the AP isomer is found only slightly higher in energy (<2 kJ mol^−1^) and therefore possibly both isomers may be populated in the experiment (see ESI Fig. S10–S12†). Like for neutral *i*-PrOH, the SP isomer represents a first-order transition state and lies up to 4 kJ mol^−1^ higher in energy than the two symmetry-equivalent SC isomers. The lower energy SC isomer of X^−^(*i*-PrOH) exhibits a shorter and hence stronger IHB than the AP isomer. In general, the IHB in X^−^(*i*-PrOH) is roughly 5–10% longer compared to that in X^−^(HFIP).

### 3.2 Harmonic analysis

**Fig. 3:**
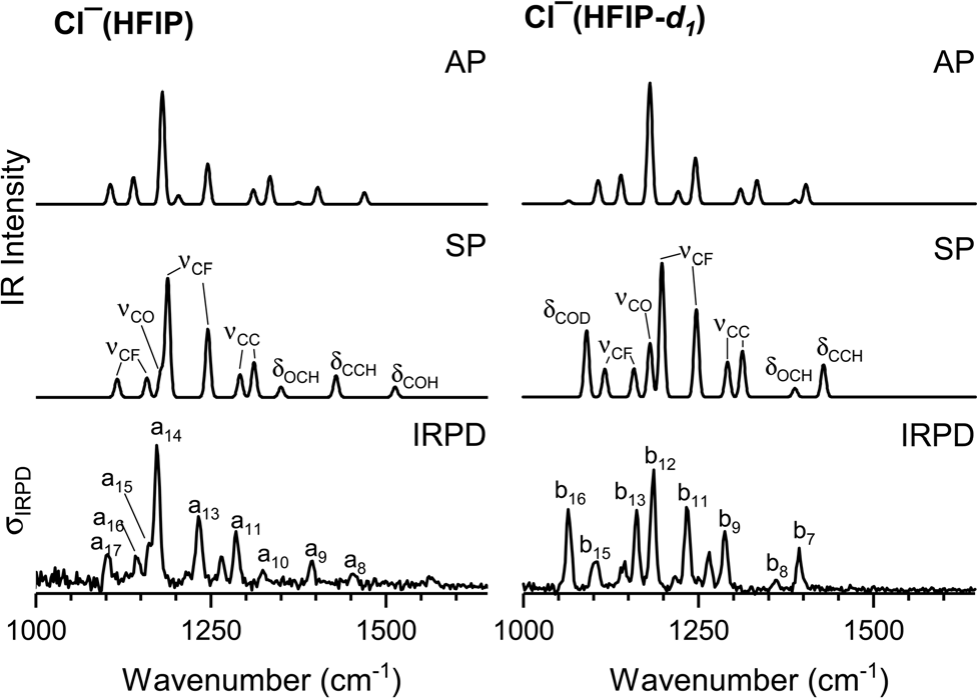
Unscaled harmonic MP2/aug-cc-pVTZ IR spectra of the AP (top panel) and the SP isomer (see Fig. 2 for geometries) of Cl^−^(HFIP) (left) and Cl^−^(HFIP-*d*_1_) (right) compared to the IRPD spectrum of the corresponding D_2_-tagged complex. See Table 3 for band positions, harmonic vibrational frequencies and assignments. The harmonic spectra were convoluted using a Gaussian line-shape function with a full width at half maximum (FWHM) of 8 cm^−1^.

In order to assign the structure of the halide anion complexes, we focus on the spectral signature in the fingerprint spectral region, which probes the excitation of the characteristic bending (*δ*_COH,_*δ*_CCH_, *δ*_OCH_) and stretching modes (*ν*_CF_, *ν*_CO_, *ν*_CC_), using the spectra of the Cl^−^(HFIP/HFIP-*d*_1_) complexes as a representative example. The (unscaled) harmonic IR spectra predicted for the SP and the AP isomers of Cl^−^(HFIP/HFIP-*d*_1_) in the fingerprint region are shown in Fig. 3 (see Fig. S4 and S6 in the ESI† for a comparison of the corresponding spectra of the Br^−^ and I^−^ complexes), where they are compared to the IRPD spectrum of the corresponding D_2_-tagged complex (same spectra as in Fig. 1). Band positions, harmonic frequencies and band assignments are summarized in Table 3. Satisfactory agreement between the harmonic spectrum and the experimental IRPD spectrum is only found for the SP isomers in all cases, not only with respect to the vibrational frequencies, but also with respect to the relative intensities (see Fig. 3), consolidating the notion that we observe the lowest energy isomer and can indeed assign most of the IRPD bands accordingly (see Table 3).

**Table 3:** Band labels, X^−^(HFIP)·D_2_ IRPD band positions (in cm^−1^), X^−^(HFIP) harmonic MP2/aug-cc-pVTZ vibrational frequencies (in cm^−1^) and band assignments of the fundamental transitions for X^−^ = Cl^−^, Br^−^ and I^−^. Values for the corresponding deuterated isotopologue are given in parentheses

Cl^−^			Br^−^			I^−^			Assignment^a^
Label	IRPD	MP2	Label	IRPD	MP2	Label	IRPD	MP2	
a_8_ (b_16_)	1453 (1064)	1513 (1090)	(d_15_)	- (1063)	1498 (1079)	(f_18_)	- (1041)	1482 (1062)	*δ*_COH_ (*δ*_COD_)
a_9_ (b_7_)	1395 (1394)	1429 (1428)	c_12_ (d_6_)	1392 (1394)	1430 (1430)	e_10_ (f_8_)	1395 (1394)	1429 (1429)	*δ*_CCH_
a_10_ (b_8_)	1324 (1360)	1350 (1388)	c_13_ (d_7_)	1313 (1364)	1346 (1390)	e_11_ (f_9_)	1319 (1369)	1338 (1395)	*δ*_OCH_
a_11_ (b_9_)	1286 (1287)	1311 (1313)	c_14_ (d_8_)	1285 (1289)	1313 (1315)	e_12_ (f_10_)	1290 (1291)	1314 (1317)	*ν*_CC_
a_12_ (b_10_)	1264 (1265)	1291 (1291)	c_15_ (d_9_)	1263 (1267)	1291 (1291)	e_13_ (f_11_)	1267 (1265)	1291 (1291)	*ν*_CC_
a_13_ (b_11_)	1233 (1233)	1246 (1247)	c_16_ (d_10_)	1234 (1235)	1247 (1248)	e_14_ (f_12_)	1238 (1237)	1249 (1250)	*ν*_CF_
a_14_ (b_12_)	1173 (1186)	1188 (1198)	c_17_ (d_11_)	1175 (1187)	1190 (1200)	e_16_ (f_14_)	1177 (1190)	1191 (1203)	*ν*_CF_
a_15_ (b_13_)	1162 (1162)	1179 (1181)	c_18_ (d_12_)	1154 (1155)	1174 (1175)	e_17_ (f_15_)	1152 (1152)	1169 (1169)	*ν*_CO_
a_16_ (b_14_)	1144 (1143)	1159 (1158)	c_19_ (d_13_)	1141 (1145)	1161 (1161)	e_18_ (f_16_)	1137 (1134)	1164 (1164)	*ν*_CF_
a_17_ (b_15_)	1102 (1104)	1116 (1116)	c_20_ (d_14_)	1103 (1108)	1118 (1118)	e_19_ (f_17_)	1107 (1107)	1120 (1120)	*ν*_CF_

a Assignment to local stretching (*ν*) and bending (*δ*) vibrational modes. See ref. 31 for a detailed description of modes in neutral HFIP molecule.

**Fig. 4:**
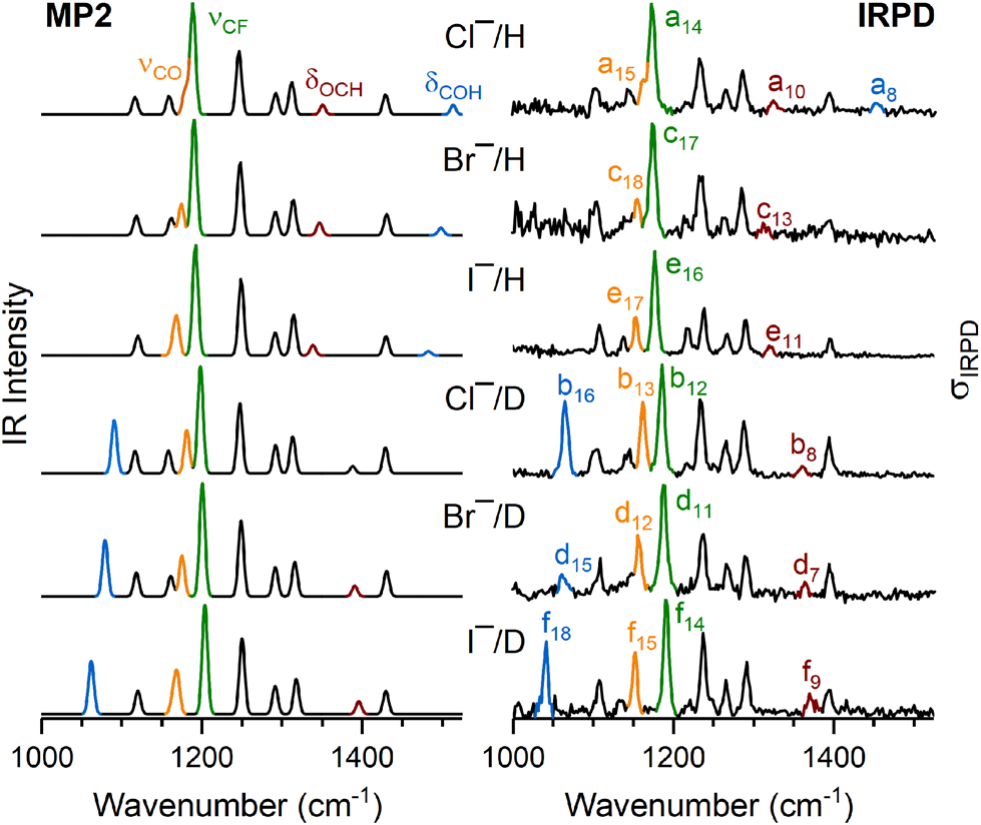
MP2/aug-cc-pVTZ spectra (left) of the untagged complexes compared to IRPD spectra of D_2_-tagged X^−^(HFIP) and X^−^(HFIP-*d*_1_) (right) in the spectral region from 1000 to 1525 cm^−1^. Bands that are particularly sensitive to the nature of the halide anion are shown in color. These are the COH(D) bend (*δ*_COH(D)_, blue), the most intense CF stretch (*ν*_CF_, green), CO stretch (*ν*_CO_, orange), and OCH bend fundamental (*δ*_OCH_, red). The harmonic spectra were convoluted using a Gaussian line-shape function with a FWHM of 8 cm^−1^.

In order to visualize, how the band positions are affected by (i) deuteration and (ii) the nature of the halide anion, the unscaled harmonic IR spectra for all six HFIP-containing complexes are compared to the experimental IRPD spectra in Fig. 4.

The most obvious change predicted upon deuteration is the red-shift of the bending mode *δ*_COH_ (1513 cm^−1^) by 423 cm^−1^, which corresponds reasonably well with the experimental value of 389 cm^−1^ for the difference in band positions of a_8_ and b_16_ (blue bands in Fig. 4). In addition, two other modes, namely *δ*_OCH_ (red band) and the most IR active of the *ν*_CF_ modes (green band), which correspond to the IRPD band pairs a_10_ (b_8_) and a_14_ (b_12_), respectively, are blue-shifted upon deuteration, indicating that these modes are more delocalized than expected from a local mode picture and also sensitive to deuteration of the O–H moiety. A direct consequence of the latter shift is that the excitation of the CO stretching mode, *ν*_CO_, (orange bands in Fig. 4), which appears as a shoulder at 1162 cm^−1^ (a_15_) in the IRPD spectrum of Cl^−^(HFIP), is clearly visible as an isolated band (b_13_) in the IRPD spectrum of Cl^−^(HFIP-*d*_1_).

The spectra for the different halide anions look very similar, the observed effects are small and the agreement between the predicted and experimental spectra remains satisfactory. Small spectral red-shifts (with increasing halide anion size) are predicted and observed for the excitation of the CO stretching mode, *ν*_CO_, (Cl^−^: 1162 cm^−1^, Br^−^: 1155 cm^−1^, I^−^: 1153 cm^−1^), and the in-plane COD bending mode, *δ*_COD_, (Cl^−^: 1090 cm^−1^, Br^−^: 1063 cm^−1^, I^−^: 1041 cm^−1^). Both can be rationalized on the basis of the decreasing HB strength with increasing halide anion size, which results in a stronger O–H bond and hence slightly weaker C–O bond in the first case, and a longer heavy atom distance and hence a weaker cage effect, in the second case.

### 3.3 Anharmonic analysis

Vibrational frequencies of hydroxyl groups involved in IHBs are not well reproduced within the harmonic approximation^24,27,32,33^ and we therefore calculated OH stretching frequencies considering anharmonic effects using two complementary methods (see Table 1). VPT2 is a well-established method to calculate anharmonic corrections and implemented many quantum chemistry packages.^34^ However, problems occur when accidental degeneracies are encountered.^35^ This is increasingly the case for larger systems, but also well documented for smaller systems in the OH stretching region, where the first overtone as well as combination bands involving various bending modes can contribute to Fermi resonances with the *ν*_OH_ fundamental.^24,32^ To overcome such shortcomings, a variational approach is necessary to be considered and we do this using the DVR-FBR technique (see methods).^36–38^

**Fig. 5:**
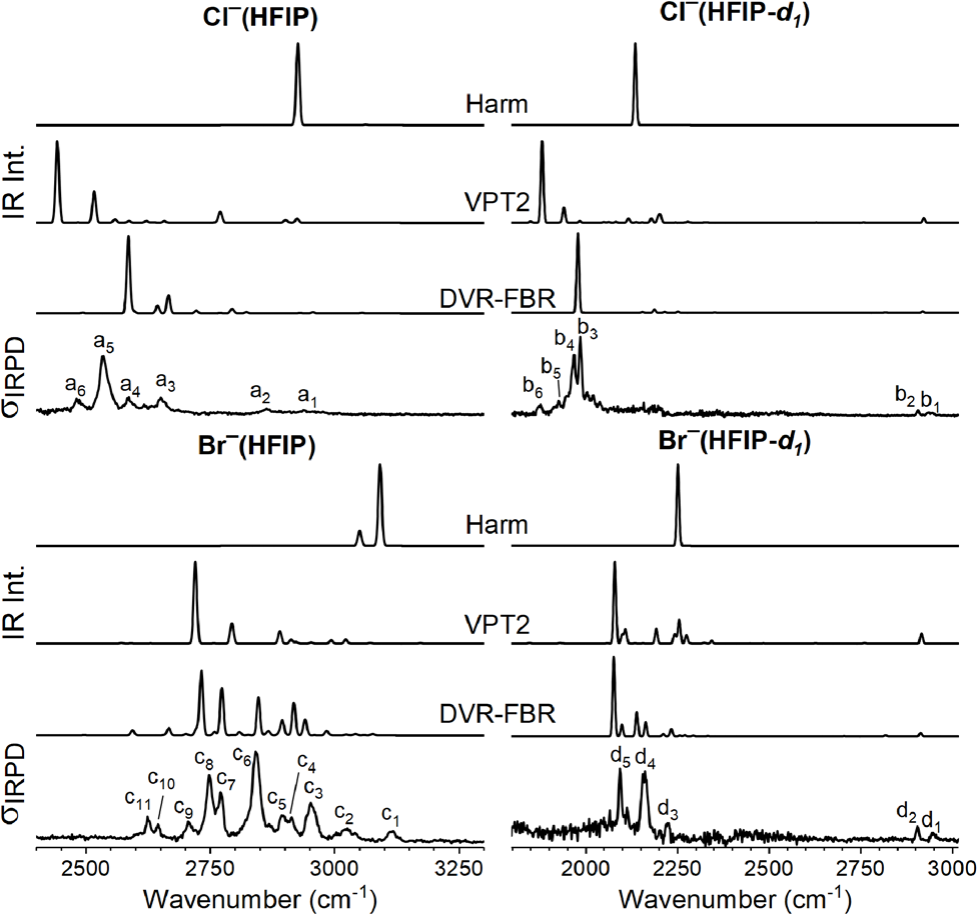
Simulated IR spectra based on anharmonic frequencies and intensities from vibrational second order perturbation theory (VPT2) and discrete variable representation with finite basis representation (DVR-FBR) calculations of X^−^(HFIP) (left) and X^−^(HFIP-*d*_1_) (right), X^−^ = Cl^−^ (top half), Br^−^ (bottom half), compared to IRPD spectrum of the corresponding D_2_-tagged complex. Harmonic, VPT2 and DVR-FBR spectra were convoluted using a Gaussian line-shape function with a FWHM of 8 cm^−1^.

The predicted IR spectra including anharmonic effects are compared to the IRPD spectra in the OH(D) stretching region for Cl^−^(HFIP/HFIP-*d*_1_) and Br^−^(HFIP/HFIP-*d*_1_) in Fig. 5. While the (unscaled) harmonic frequencies substantially overestimate the experimental values for *ν*_OH(D)_ (see Table 1), VPT2 systematically underestimates these, except for Br^−^(HFIP-*d*_1_). Apart from excitation of the *ν*_OH(D)_ fundamental, the most intense features predicted by the VPT2 method are combinations of *ν*_OH(D)_ with low-frequency, large amplitude ion-molecule modes. In contrast, the first overtone transitions of the *δ*_COH,_*δ*_CCH_, and *δ*_OCH_ bending modes are predicted weak in intensity. Overall, the agreement of the VPT2 spectra with the IRPD spectra in the OH(D) stretching is improved, compared to the harmonic analysis, but is not as good as with DVR-FBR method, discussed below.

To gain a more quantitative understanding of the nature and extent of anharmonic effect, in particular, to elucidate the role of Fermi resonances in the OH(D) stretching region more reliably, we performed DVR-FBR calculations. For the X^−^(HFIP) complexes, we included five stretching modes (*ν*_CH_, *ν*_OH(D)_, *ν*_CO_, *ν*_CC_, and *ν*_OH(D)⋯X_) and four bending modes (in-plane *δ*_COH(D)_, *δ*_CCH_, *δ*_OCH_, and out-of-plane 
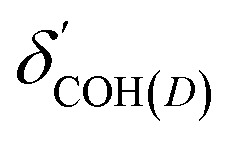
); for the X^−^(HFIP-*d*_1_) complexes, we choose the nine modes above plus one additional CC stretching mode 
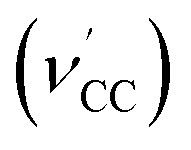
. It should be noted that although we use same naming convention as for the harmonic calculations, the *δ*_CCH_, *δ*_OCH_, *δ*_COD_ and 
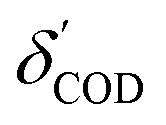
 bending modes are well-separated in the case of X^−^(HFIP-*d*_1_); in contrast, for X^−^(HFIP), their counterpart mix strongly with each other. In our experience, the HB stretching mode *ν*_OH⋯X_ usually plays an important role in 1-to-1 complexes, but the harmonic analysis usually separates its contribution into many low-frequency normal modes. Therefore, we adopt the “intermolecular translation mode”^39^ to represent the contribution from the HB stretching motion.

The satisfactory agreement between the DVR-FBR and corresponding IRPD spectra in Fig. 5 shows that excitation of the *ν*_OH(D)_ fundamental as well as many two and three-quanta states involving excitation of *ν*_OH(D)_ in combination with the above-mentioned modes is well described (see Table 4 for band assignments). For Br^−^(HFIP), the two most intense peaks in the DVR-FBR spectrum are predicted at 2731 cm^−1^ and 2772 cm^−1^. These transitions contain the highest contribution of *ν*_OH_. However, the relative weight of *ν*_OH_ is only 0.20 and 0.16, respectively, indicating the extend of anharmonic coupling in this particular system, which is also evidenced by pronounced intensity borrowing of several of the two/three-quanta states located between 2600 to 3000 cm^−1^. In the case of Cl^−^(HFIP), the “*ν*_OH_ fundamental” is shifted to be below 2600 cm^−1^, so two-quanta states above 2700 cm^−1^ do not gain much intensity due to detuning. The most visible feature in this case is the doublet at 2588 cm^−1^ and 2665 cm^−1^ assigned to *ν*_OH_ and *δ*^2^_OCH_, respectively. For Cl^−^(HFIP-*d*_1_) and Br^−^(HFIP-*d*_1_), the latter has more complex vibrational feature than the former also due to better resonance condition between *ν*_OD_ and the two/three-quanta states. Since these two/three-quanta states are heavily mixed, we only list the leading components in Table 4, to aid the band assignments.

**Table 4:** Band labels, IRPD band positions (in cm^−1^), DVR-FBR anharmonic frequencies and band assignments (*ν*: stretching mode, *δ*: bending mode) for Cl^−^(HFIP), Cl^−^(HFIP-*d*_1_), Br^−^(HFIP) and Br^−^(HFIP-*d*_1_) based on the results of the DVR-FBR calculations. Only the assigned IRPD features are listed

System	Label	IRPD	DVR-FBR	Leading components
Cl^−^(HFIP)	a_1_	2936	2929	0.28 *ν*_CH_
	a_3_	2652	2665	0.47 *δ*^2^_OCH_, 0.10 *ν*_OH_
	a_4_	2585	2643	0.68 *ν*_CO_ + *δ*_COH_
	a_5_	2535	2584	**0.58 *ν***_**OH**_
	a_7_	1566	1616	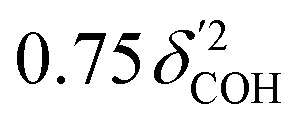
				
Cl^−^(HFIP-*d*_1_)	b_1_	2935	2919	0.65 *ν*_CH_
	b_3,_ b_4_	1975	1977	**0.80 *ν***_**OD**_
				
Br^−^(HFIP)	c_3_	2953	2940	0.55 *ν*_CH_
	c_5_	2896	2894	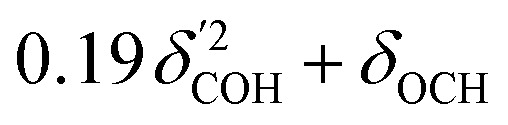
	c_6_	2841	2846	0.24 *δ*_OCH_ + *δ*_COH_, 0.10 *ν*_OH_
	c_7_	2770	2772	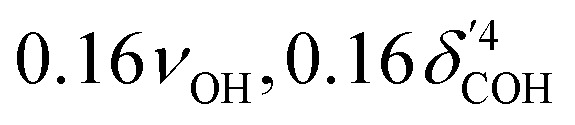
	c_8_	2748	2731	0.37 *δ*_OCH_ + *δ*_COH_, **0.20 *ν***_**OH**_
	c_9_	2707	2665	0.46 *ν*_CC_ + *δ*_OCH_
	c_11_	2623	2593	0.94 *ν*_CO_ + *δ*_COH_
				
Br^−^(HFIP-*d*_1_)	d_1_	2945	2913	0.61 *ν*_CH_
	d_3_	2222	2232	0.64 *ν*_CO_ + *δ*_COD_
	d_4_	2161	2163	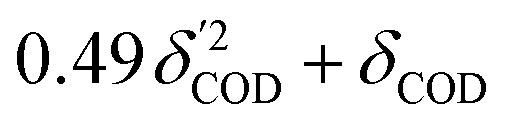
			2138	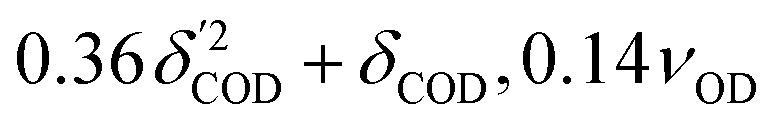
	d_5_	2092	2075	**0.51 *ν***_**OD**_

### 3.4 Bonding analysis

After obtaining a satisfactory assignment of the IRPD spectra, we now move our focus to gaining a better understanding of the interactions at play in the X^−^(HFIP/HFIP-*d*_1_) complexes, in particular, how the observed red-shift Δ*ν*_OH_ is related to the stability of the complex. Note, the most stable species, which is also the one we observe in the experiment, contains HFIP in its SP configuration. However, this isomer is predicted to exhibit a smaller red-shift and hence a weaker and longer O–H⋯X^−^ IHB (see Table 2) than the one containing HFIP in the AP configuration. In the following we will employ the Morokuma-Ziegler energy decomposition analysis (EDA) method^40–43^ in combination with natural orbitals for chemical valence (NOCV) extension,^44^ to quantify the bonding contributions and to derive trends.

Since the EDA-NOCV has been developed for DFT-based approaches, we conduct the analysis with B3LYP-D3(BJ)/TZ2P,^45–48^ which has been found to accurately reproduce the structures from the MP2 approach outlined above (see ESI, Table S4† for comparison of energies and geometrical parameters). In Table 5, we summarise the main findings for the Cl^−^ complex. Similar findings for X = Br^−^ and I^−^ can be found in the ESI (Tables S5 and S6†), the only notable trend being the decreasing bond strength as the halide anion increases in size (Cl^−^ > Br^−^ > I^−^).

**Table 5:** EDA-NOCV results^a^ of HBs between H_2_O, HFIP, *i*-PrOH and the chloride anion

	Cl^−^(HFIP)_SP_		Cl^−^(HFIP)_AP_		Cl^−^(*i*-PrOH)		Cl^−^(H_2_O)	
Δ*E*_int_	−164		−134		−84		−73	
Δ*E*_int_(disp)^b^	−9	(5%)	−9	(7%)	−10	(12%)	−4	(5%)
Δ*E*_int_(elec)^b^	−155	(95%)	−125	(93%)	−74	(88%)	−69	(95%)
Δ*E*_Pauli_	+111		+120		+75		+55	
Δ*E*_elstat_^c^	−171	(64%)	−140	(57%)	−87	(58%)	−82	(66%)
Δ*E*_orb_^c^	−95	(36%)	−106	(43%)	−63	(42%)	−43	(34%)
Δ*E*_1_(Cl^−^ → H–O)^d^	−61	(64%)	−72	(68%)	−33	(53%)	−32	(74%)
Δ*E*_2_(Cl^−^ → H–C)^d^	−9	(9%)						
Δ*E*_3_(Cl^−^ → H–C_Me_)^d^					−8	(12%)		
					−5	(8%)		
Δ*E*_prep_	+22		+21		+4		+3	
*E*_bond_	−142		−113		−80		−70	
*d*(Cl^−^–H)	193		187		213		212	

a Energies in kJ mol^−1^ and bond length in pm.

b Percentage values give the relative contributions of dispersion and electronic effects to Δ*E*_int_.

c Percentage values give the relative contributions to the attractive EDA terms Δ*E*_elstat_ and Δ*E*_orb_.

d Percentage values give the relative contributions of the NOCV to Δ*E*_orb_.

First, we find that the bond strength ordering as a function of the ligand is H_2_O < *i*-PrOH ≪ HFIP with HFIP showing more than twice the bond energy (*E*_bond_) compared to H_2_O. This is also reflected in the interaction energy (Δ*E*_int_), although HFIP shows a higher deformation upon HB formation reflected in a sizeable preparation energy (Δ*E*_prep_ = 22 kJ mol^−1^) – a term which is close to zero for H_2_O and *i*-PrOH. This is a result of the configurational change from the AP to the SP isomer (see Fig. 2). The SP isomer shows additional stabilization due to a second (weaker) HB involving the CH group. A structural indicator of this second HB is the deviation of the HB angle (*θ*_OHX_ = 163°) from the ideal linear configuration. However, the small increase of 10 pm in the C–H bond length suggests that the second HB is much weaker.

The first notable observation upon decomposing the bond energy is that the dispersion energy contribution, Δ*E*_int_(disp), is nearly negligible for all complexes listed in Table 5. Although taking the DFT-D3 term as indicator of the attractive London forces is an approximation, the low value compared to the covalent bonding contribution, Δ*E*_int_(elec), is a strong indicator that the ion-molecule interaction is not governed by dispersion attraction. The EDA procedure clearly shows the cause for the stronger interaction in the most stable complex Cl^−^(HFIP)_SP_ compared to the non-fluorinated alcohol *i*-PrOH complexes: the electrostatic attraction term (Δ*E*_elstat_) is considerably larger in its absolute terms (+84 kJ mol^−1^) as well as in its relative contribution (+6%). Due to the shorter HB, the other two EDA terms (Δ*E*_Pauli_, Δ*E*_orb_) are also larger in HFIP compared to *i*-PrOH complex but the term dominating the trend is decisive here. This makes the HB in the HFIP complex more similar to that in the H_2_O complex, where the electrostatic term is also the most important attractive interaction.

For the higher energy Cl^−^(HFIP)_AP_ isomer several characteristic differences are found (Table 5). Even though the HB is shorter, the total interaction energy is smaller. This can be mainly traced back to a significant decrease in electrostatic attraction due to a less favorable dipole–ion interaction in the Cl^−^(HFIP)_AP_ isomer. The increased orbital term points towards a larger charge-transfer and hence also more pronounced red-shift Δ*ν*_OH_ (see ESI Fig. S3–S6†). However, this increase is not sufficient to compensate for the smaller electrostatic interaction. The SP isomer is further stabilized by a second, albeit considerably weaker, X^−^⋯H–C HB, which is not present in the AP isomer (Δ*E*_2_ in Table 5).

**Fig. 6:**
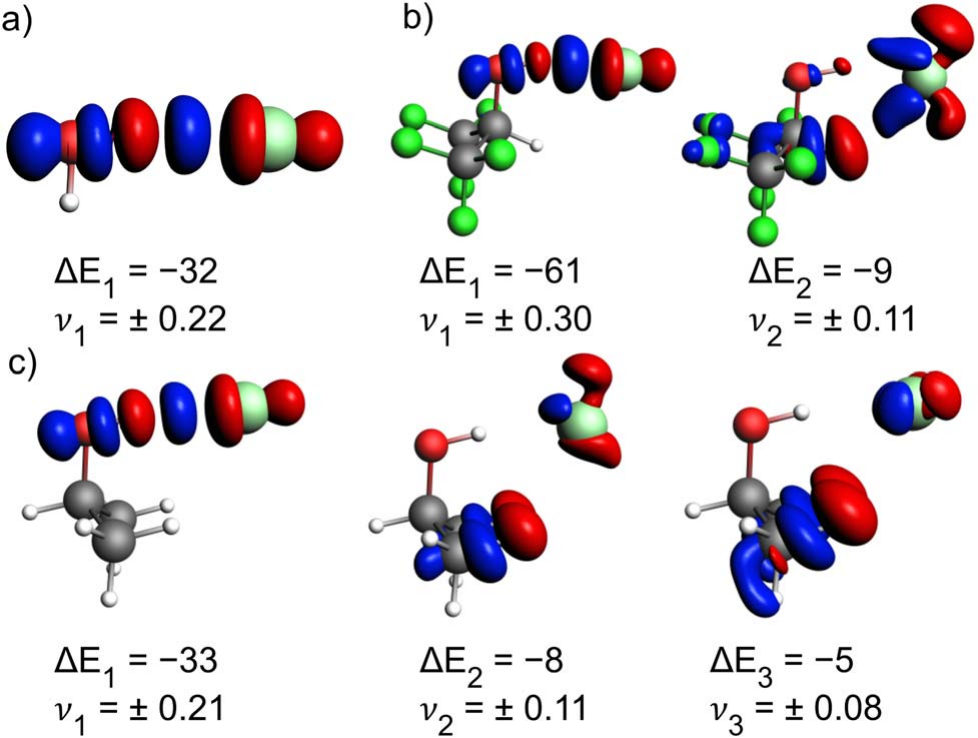
Selected deformation densities (Δ*ρ*_*i*_) from EDA-NOCVs with energy contribution (Δ*E*_*i*_, further explained in Table 5) to Δ*E*_orb_ in kJ mol^−1^ and eigenvalues (*ν*_i_). Charge depletion (red) and charge accumulation (blue) for (a) Cl^−^(H_2_O), (b) Cl^−^(HFIP) and (c) Cl^−^(*i*-PrOH). Iso values are chosen for clarity.

The deformation densities from the NOCV analysis (Fig. 6) show the most important orbital interactions contributing to Δ*E*_orb_. In all three cases, the major contribution is the donation from a non-bonding Cl^−^ lone pair orbital into the antibonding σ*(O–H) orbital (Δ*E*_1_). Notably, this interaction is of similar magnitude in the H_2_O (Fig. 6a) and *i*-PrOH complexes (Fig. 6c), but nearly twice as strong in the HFIP SP-complex (Fig. 6b) with Δ*E*_1_ = −61 kJ mol^−1^. The NOCV analysis also gives a hint towards the strength of the secondary HB interaction, which only appears in the alcohol interacting with Cl^−^. This interaction is much weaker but still accounts for 9 kJ mol^−1^ in the HFIP complex (Fig. 6b, Δ*E*_2_). For the *i*-PrOH complex, two weak C_Me_–H⋯Cl HBs are found with 8 and 5 kJ mol^−1^ orbital interaction contribution, respectively (Fig. 6c). We thus conclude from the present bonding analysis that, similar to the X^−^(H_2_O) complexes, the interactions in the X^−^(HFIP) complexes are dominated by electrostatic attraction, which overrules the trends from charge transfer effects. Dispersion attraction only plays a minor and non-decisive role. The electrostatic attraction is largest in the SP isomer and hence this represents the most stable complex, even though the X^−^⋯H–O HB interaction is weaker than in the higher energy AP isomer.

## 4 Discussion

The above analysis demonstrates that the electrostatic attraction is the dominant term contributing to the strength of the IHB in the halide anion complexes discussed here, but it is not directly correlated to the observed red-shift Δ*ν*_OH_. Similarly, a water molecule (1.85 D)^49^ has a slightly large dipole moment compared to *i*-PrOH (1.58 D),^50^ but the corresponding red-shift Δ*ν*_OH_ of the X^−^(H_2_O) (see Table 1) is slightly smaller than that of X^−^(*i*-PrOH), because the X^−^(H_2_O) complexes do not adopt the “dipolar” *C*_2v_ geometry, which optimizes the electrostatic attraction term, but rather a *C*_s_ one with a quasi-linear IHB. The driving force for this symmetry breaking is the orbital interaction in the form of charge transfer from the anion to H_2_O's antibonding σ* orbitals, which is maximized for a linear IHB. Indeed, it is this charge transfer component that manifests itself as a red-shift in the OH stretching vibrational frequency associated with a IHB.^18^

The seminal work on the vibrational spectroscopy of X^−^(H_2_O) complexes by Johnson and coworkers^21^ revealed that the vibrational red-shift Δ*ν*_OH_ is indeed correlated with the halide anion proton affinity (PA). This confirmed the predictions by Thompson and Hynes based on a two valence-bond (VB) state model, in which the first VB state has the charge character X^−^⋯H_2_O and the second is a charge-transfer VB state with electronic structure XH⋯OH^−^.^19^ Δ*ν*_OH_ is governed by the relative energy of the XH⋯OH^−^ diabatic state, which correlates with the, PA of the anion.^51^

The PA is defined as the negative enthalpy 
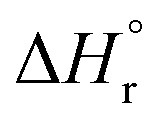
 of the gas phase reactionX + H^+^ → XH^+^,where X can be electrically neutral or not and H^+^ represents a hydron.^52^ Plotting Δ*ν*_OH_ as a function of the anion PA then yields a monotonically increasing function with a rather linear behaviour for smaller red-shifts and a positive curvature at higher red-shifts.^21^ However, a simple understanding of this behaviour is not evident.^19^

In order to extend this model to different solvent molecule, the above reaction can be rewritten asX^−^ + HM → XH + M^−^.We now also need to consider the proton donor ability of the neutral molecule (HM), namely, its deprotonation enthalpy, which corresponds to the negative value of the PA of the conjugate base M^−^. The difference in the proton affinities (ΔPA),

2 ΔPA = PA(X^−^) − PA(M^−^),

should then reflect extent of charge transfer and hence also correlate with Δ*ν*_OH_.

**Fig. 7:**
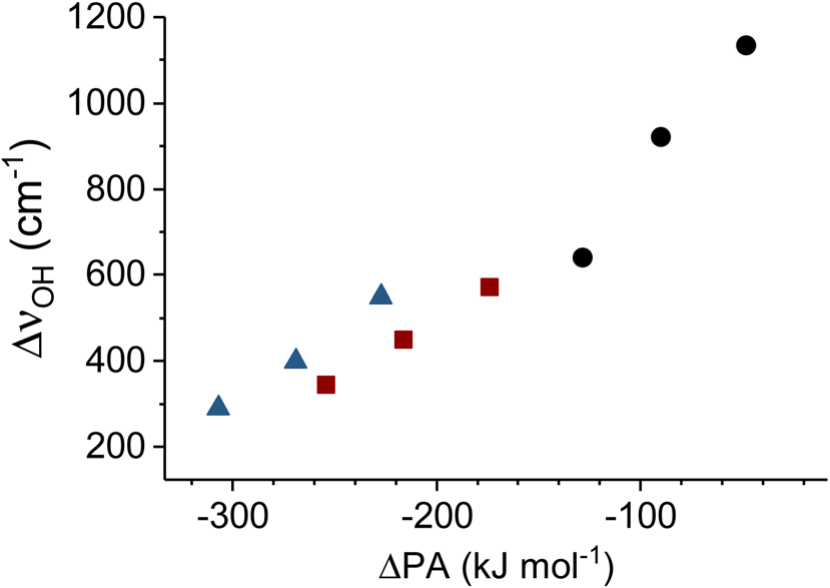
Red-shift of the OH stretching frequency (Δ*ν*_OH_) associated with the IHB as a function of the difference in proton affinities ΔPA (see eqn (2)) associated with the X^−^(HM) complexes with X^−^ = Cl^−^, Br^−^, I^−^ and HM = HFIP (circles), *i*-PrOH (squares), H_2_O (triangles).

The ΔPA values for the systems studied here are listed in Table 6 and Δ*ν*_OH_ is plotted against ΔPA in Fig. 7. Several interesting observations can be made. First, the set of red-shifts observed for a particular neutral molecule, and also for each halide anion, are consistent in that an increase in ΔPA leads to an increase Δ*ν*_OH_. However, the overall agreement is less satisfactory. In more detail, the red-shifts observed for the H_2_O and *i*-PrOH complexes are similar, even though H_2_O exhibits a considerably larger PA. Hence, the extent of charge transfer does not only depend on the relative energy of the VB states (as we are assuming here), but also on other parameters, like the HB angle *θ*_OHX_ (see Table 2). The IHB in the water complexes are nearly linear, while they deviate substantially from linearity in the isopropanol (∼165°) and the HFIP (∼160°) complexes, reducing the orbital overlap with the 
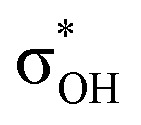
 orbitals and consequently the amount of charge transfer.

**Table 6:** ΔPA values (in cm^−1^) for the X^−^ + HM → XH + M^−^ reaction from experimentally determined proton affinities^53^

X^−^\HM	H_2_O	*i*-PrOH	HFIP
Cl^−^	−227	−174	−48
Br^−^	−269	−216	−90
I^−^	−307	−254	−128

The transitions assigned to the OH stretching fundamentals are characterized by substantial mode mixing (see Table 4). To assess, in how far this affects the trends observed in Fig. 7, we compare this data to the corresponding data for the deuterated species in Fig. S15 (see ESI).† The extent of mode mixing is substantially reduced upon deuteration, as evidenced, for example, by the considerably simpler IRPD spectra of the deuterated species. Except for the expected reduction in the absolute red-shift upon deuteration, there is no qualitative difference between the two data sets, indicating that the effect of mode mixing only plays a minor role in the observed trends.

## 5 Conclusions

The present results confirm the original insights on the intermolecular interaction in X^−^(HFIP) complexes (X^−^ = Cl^−^, Br^−^, I^−^) reported by Wang and coworkers, based on anion photoelectron spectroscopy combined with electronic structure calculations.^16^ The halide anion interacts with the neutral HFIP *via* ionic hydrogen bonding and charge–dipole interactions, of which the latter dominate and determine the configuration of the complexes. Here we show that the interaction energy is roughly twice as large as in the corresponding complexes with H_2_O and *i*-PrOH, confirming that HFIP is a superior HB donor. The reported vibrational frequency red-shifts yield detailed insight into the role of charge transfer in these complexes, which follow a similar trend as the interaction energy. To obtain an accurate energy balance the contribution from the weaker X^−^⋯H–C HB also needs to be considered.

While the vibrational transitions in the fingerprint region are well reproduced within the harmonic approximation, the reliable prediction of the features in the OH(D) stretching region require an anharmonic treatment. DVR calculations allow evaluating the contribution of overtones and combination bands involving various bending modes to Fermi resonances in the O–H stretching region. These insights emphasize the relevance of anharmonic methods that go beyond standard approaches like VPT2 for a reliable prediction of the signal carrier, whenever ions are present, for example, in electrochemical applications.

Finally, we propose a generalized model for qualitatively predicting the vibrational frequency red-shift Δ*ν*_OH_ based on the difference in the proton affinities of the two conjugated base anions of a proton transfer reaction. This model qualitatively reproduces the observed trends, in particular, when the differences in the HB geometries are small.

## 6 Methods

### 6.1 Experimental methods

IRPD spectroscopic experiments were performed using a cryogenically cooled ion trap triple mass spectrometer described elsewhere.^20^ In brief, anion–molecule complexes are produced in a nanospray ion source from 0.1–0.5 mM sodium halide solutions (NaCl: Sigma-Aldrich, ≥99.0%; NaBr: Merck, extra pure; NaI: Sigma-Aldrich, 98%) in either ACN/H_2_O (1 : 2, v/v), H_2_O/*i*-PrOH (1 : 10, v/v) or 0.5 mM HFIP in MeOH/H_2_O (1 : 2, v/v). Partially deuterated complexes were obtained by H/D exchange in the gas phase,^54^ except for *i*-PrOH, for which a solution containing *i*-PrOD, D_2_O and D_2_SO_4_ was used. Typical mass spectra of these solutions are shown in Fig. S1 and S2 of the ESI.†

The beam of anions is skimmed, collimated in a gas-filled radio frequency (RF) quadrupole ion guide, mass-selected using a quadrupole mass-filter and focused in a RF ring-electrode ion trap, held at a temperature of 12–14 K and continuously filled with D_2_ gas. Many collisions of the trapped ions with the buffer gas provide gentle cooling of the internal degrees of freedom close to the ambient temperature. At sufficiently low ion-trap temperatures, ion–messenger complexes are formed *via* three-body collisions.^55^ Every 100 ms, all ions are extracted from the ion trap and focused, both temporally and spatially, into the centre of the extraction region of the orthogonally-mounted double-focussing reflectron time-of-flight (TOF) tandem photofragmentation mass spectrometer and detected using the background-free IR^1^MS^2^ detection scheme.^56^ To this end, the ion packet is accelerated into the reflectron stage. Ions spread out in space according to their mass-to-charge ratio (*m*/*z*) and are refocused at the initial extraction region. Prior to be reaccelerated towards the MCP detector, ion–messenger complexes with a particular *m*/*z* value are irradiated by a properly timed and widely wavelength tunable IR laser pulse (bandwidth: 3.5 cm^−1^). The IR pulse is supplied by an optical parametric oscillator/amplifier (LaserVision: OPO/OPA/AgGaSe_2_) laser system pumped by an unseeded Nd:YAG laser (Continuum Surelite EX).^57^ IRPD spectra are recorded by monitoring the intensity of the irradiated ions and their photofragments while the laser wavelength is monitored online using a HighFinesse WS6-600 wavelength meter. The wavelength scanned continuously with a scan speed such that an averaged TOF mass spectrum (over 60 laser shots) is obtained every 2 cm^−1^. Typically, three to five scans are measured and averaged and the photodissociation cross section *σ*_IRPD_ is determined as described previously.^20,58^

### 6.2 Computational methods

#### 6.2.1 Energetics and harmonic analysis

Electronic structure calculations were performed using the Gaussian 16 rev. C01 program package.^59^ Geometry optimizations followed by harmonic vibrational frequency calculations were performed using second-order Møller–Plesset perturbation theory (MP2)^60^ in combination with either the aug-cc-pVDZ (aug-cc-pVDZ-PP for iodine) or the aug-cc-pVTZ (aug-cc-pVTZ-PP for iodine) basis set.^61^ Electronic energies, minimum-energy structures, and harmonic frequencies are found at 10.5281/zenodo.14361478. Simulated IR spectra were obtained by convolution of the vibrational stick spectra with a Gaussian line shape function with a full width at half maximum of 8 cm^−1^ to account for the bandwidth of the IR laser pulse, rovibrational excitation, as well as the predissociation lifetime.

#### 6.2.2 Anharmonic analysis

Two different approaches were carried out to account for the effect of vibrational anharmonicities on the IR spectra. First, we used standard vibrational perturbation theory (VPT2).^34^ The full MP2/aug-cc-pVDZ (or aug-cc-pVDZ-PP for iodine) potential energy surface (PES), truncated at quartic terms, was calculated to evaluate the relative energies and IR intensities of fundamental and two quanta states of all normal modes. We refer to this approach as VPT2/MP2/aug-cc-pVDZ (or just VPT2).

Second, to account for higher order terms in the PES and dipole moment surface (DMS), we applied *ab initio* anharmonic algorithms developed in previous works^37^ by some of us in which the PES and DMS along the selected modes were scanned on the discrete variable representation (DVR) quadrature, and the Hamiltonian matrix can be diagonalized to obtain eigenstates. The IR absorption intensities were obtained from the eigenvectors and the DMS accordingly. To construct the PES (and DMS), single-point energy (and dipole) calculations at grid points generated by the Gauss–Hermite quadrature were performed along the selected vibrational modes; the PES is scanned with at the level of RI-MP2/aug-cc-pVTZ with corrections for CH and OH(OD) stretching modes at the level of DLPNO-CCSD(T)/aug-cc-pVTZ.^37^ The single point calculations for PES and DMS were performed with the ORCA program package.^62^ Since DLPNO-CCSD(T)/aug-cc-pVTZ is not applicable to I^−^, we only simulated complexes with X^−^ = Cl^−^, Br^−^. The total number of grid points is quite large to diagonalize the Hamiltonian directly; therefore, we solve the Hamiltonian by transforming it into Finite-Basis-Representation (FBR).^38^ We refer to this approach as DVR-FBR/RI-MP2+DLPNO-CCSD(T)/aug-cc-pVTZ (or just DVR-FBR). The detail of the methodology, and the comparison between these two methods are discussed in the ESI.†

#### 6.2.3 Bonding analysis

The HB between HFIP, *i*-PrOH, H_2_O, and halide anions was analyzed by the Morokuma–Ziegler energy decomposition analysis method (EDA).^40–43^ EDA splits the system into fragments and results in a quantitative analysis of the following bonding contributions: preparation energy (deformation of fragments for bonding), dispersion interaction, electrostatic attraction, Pauli repulsion and orbital interaction (charge transfer and polarization). The EDA computations were done with AMS, version 2021.105 (ref. 63) using B3LYP^45,47^ with a TZ2P basis set.^46^ Further information is found in the ESI.†

## Data availability

The data supporting this article have been included as part of the ESI.† Additional data from electronic structure calculation are openly available in Zenodo at: https://doi.org/10.5281/zenodo.14361478.

## Author contributions

MB: conceptualization, investigation, data curation, formal analysis, writing – original draft, writing – review & editing. FEN: investigation, data curation. JJ: conceptualization, data curation, formal analysis, software, supervision, writing – review & editing. KRA: conceptualization, funding acquisition, supervision, project administration, writing – original draft and writing – review & editing. FK: investigation, data curation, formal analysis, writing – original draft, writing – review & editing. RTZ: conceptualization, supervision, project administration, writing – original draft and writing – review & editing. QRH: investigation, data curation, formal analysis, writing – review & editing. JLK: conceptualization, supervision, writing – review & editing.

## Conflicts of interest

There are no conflicts to declare.

## Supplementary Material

SC-OLF-D4SC08456J-s001
