# Supplementary material for: Quantifying hexafluoroisopropanol's hydrogen bond donor ability: infrared photodissociation spectroscopy of halide anion HFIP complexes
Source: Chem Sci. 2025 Jan 29;16(12):5174–85. doi: 10.1039/d4sc08456j (PMC11838612; doi:10.1039/d4sc08456j)
Supplement: SC-016-D4SC08456J-s001 [file SC-016-D4SC08456J-s001.pdf]

Supplementary Information (SI) for Chemical Science.
This journal is © The Royal Society of Chemistry 2025

Supporting Information

Quantifying Hexafluoroisopropanol's Hydrogen Bond Donor Ability: Infrared Photodissociation Spectroscopy of Halide Anion HFIP Complexes

Milena Barp, [a] Florian Kreuter,[a] Qian-Rui Huang,[b] Jiaye Jin,[a] Franka. E. Ninov,[a] Jer-Lai Kuo,*[b] Ralf Tonner-Zech*[a] and Knut R. Asmis*[a]

[a] *Wilhelm-Ostwald-Institut für Physikalische und Theoretische Chemie, Universität Leipzig, Linnéstraße 2, 04103 Leipzig (Germany)*

[b] *Institute of Atomic and Molecular Sciences, Academia Sinica No1 Roosevelt Rd, Sec 4, Taipei, 106319 Taiwan*

*E-mail: jlkuo@gate.sinica.edu.tw, ralf.tonner@uni-leipzig.de, knut.asmis@uni-leipzig.de

Table of Content

1. Methods....3
   a) Detailed DVR-FBR method....3
   b) Detailed EDA....4
2. Mass spectra....6
   a) NaX, HFIP/HFIP-*d_1*....6
   b) NaX, *i*-PrOH/ *i*-PrOD....7
3. Calculated vibrational spectra....8

3.1 Comparison of X^-(HFIP) isomers: antiperiplanar (AP) *vs* synperiplanar (SP)....8
   a) Cl^-(HFIP) and Cl^-(HFIP-*d_1*)....8
      i) DVR-FBR, OH/OD stretching vibrational transition spectral region....8
   b) Br^-(HFIP) and Br^-(HFIP-*d_1*)....9
      i) Harmonic....9
      ii) DVR-FBR, OH/OD stretching vibration transition spectral region....10
   c) I^-(HFIP) and I^-(HFIP-*d_1*)....11

3.2 X^-(HFIP), SP: Harmonic *vs.* VPT2 *vs* DVR-FBR predicted spectra....12
   a) Cl^-(HFIP) and Cl^-(HFIP-*d_1*)....12
   b) Br^-(HFIP) and Br^-(HFIP-*d_1*)....13
   c) I^-(HFIP) and I^-(HFIP-*d_1*)....14

3.3 IPRD vs Harmonic Spectra of X^-(*i*-PrOH) and X^-(*i*-PrOD)....15
   a) Cl^-(*i*-PrOH) and Cl^-(*i*-PrOD)....15
   b) Br^-(*i*-PrOH) and Br^-(*i*-PrOD)....16
   c) I^- (*i*-PrOH) and I^- (*i*-PrOD)....17
4. Tag Effect....18
   a) IRPD Br^-(HFIP) - H_2 *vs* D_2 tag....18
   b) Calculated tag effect....19
5. Comparison between EDA results and BSSE corrected MP2 dissociation energies....20
6. 5. Energy Decomposition Analysis....21
   a) Br^-(HM)....21
   b) I^-(HM)....**Fehler! Textmarke nicht definiert.**
7. Anion Proton Affinity....23
8. References....24

1. **Methods**

a) Detailed DVR-FBR method

To construct the PES (and DMS) for DVR calculation, single-point energy (and dipole) calculations at grid points generated by the Gauss−Hermite quadrature were performed along the selected vibrational modes; we use 7 grid points for CH and OH(OD) stretching modes, and 5 grid points for all the other vibrational modes. To improve efficiency, the 3-mode representation (3MR) scheme was adopted to describe the PES as follows:[1]

$$V(q_i, q_j, q_k, \ldots) = V^{(0)} + \sum_i \Delta V^{(1)}(q_i) + \sum_i \Delta V^{(2)}(q_i, q_j) + \sum_i \Delta V^{(3)}(q_i, q_j, q_k).$$

Here, $V^{(0)}$ is the potential energy at the equilibrium point, $\Delta V^{(1)}$ is the change in energy within a single normal mode, $\Delta V^{(2)}$ is the contribution from anharmonic couplings between two modes, and so on. Note that we truncated this expression at $\Delta V^{(3)}$, so any interaction among four and more modes is neglected. Furthermore, a mixed-level scheme has been used to balance accuracy and computational efficiency. Under this scheme, the most essential terms, $V^{(0)}$ to $\Delta V^{(2)}$ for CH and OH(OD) stretching modes, were calculated at the DLPNO-CCSD(T)/aug-cc-pVTZ level, and all the other terms were described by RI-MP2/aug-cc-pVTZ. The single point calculations for PES and DMS were performed with the ORCA program package.[2] Since DLPNO-CCSD(T)/aug-cc-pVTZ is not applicable to I^-, we only simulated complexes with $X^- = Cl^-, Br^-$.

The total grid points are more than three million for each case; although the 3MR approximation allows us to largely reduce the number of single point calculations, the size of the Hamiltonian does not change, thus it is still quite large to diagonalize it directly even using sparse matrix diagonalization techniques. To solve the Hamiltonian of this size, we recast the DVR Hamiltonian in the Finite Basis Representation (FBR),[3] which is easier to be truncated to a diagonalizable size. Here, we only briefly describe the method, since the method details have been reported previously.[4] The basic idea of FBR is to express the basis wavefunctions $|A_i, B_j, \ldots, C_k\rangle$ as direct product of eigenvectors of several lower-dimensional DVR Hamiltonians $\hat{H}_A$, $\hat{H}_B$, … and $\hat{H}_C$:

$$|A_i, B_j, \ldots, C_k\rangle = |A_i\rangle |B_j\rangle \ldots |C_k\rangle$$

where $|A_i\rangle$, $|B_j\rangle$, … and $|C_k\rangle$ stand for the eigenstates of $\hat{H}_A$, $\hat{H}_B$, … and $\hat{H}_C$, respectively. We used all FBR basis wavefunction whose energy is less than 15000 cm^{-1} relative to the ground state FBR basis, and we ignore any coupling between states over 12000 cm^{-1}. With these FBR basis sets, we then expand the FBR Hamiltonian and diagonalized it to obtain the final eigenstate with sparse matrix diagonalization routines in SciPy.[5]

b) Detailed EDA

The bonding energy ΔE_{bond} is then decomposed by EDA into several physically meaningful contributions that enable characterization of the chemical bond. Firstly, the bonding energy ΔE_{bond} spilt into the preparation energy ΔE_{prep} and interaction energy ΔE_{int}. The preparation energy ΔE_{prep} describes the deformation of the fragments from their optimized isolated structures to the structures in the system.

$$\Delta E_{bond} = \Delta E_{prep} + \Delta E_{int} \tag{1}$$

The interaction comprises an electronic $\Delta E_{int}(elec)$ and a dispersion contribution $\Delta E_{int}(disp)$ representing the difference in dispersion energy between the system and its fragments.

$$\Delta E_{int} = \Delta E_{int}(elec) + \Delta E_{int}(disp) \tag{2}$$

Finally, the electronic contribution $\Delta E_{int}(elec)$ can be split up into three terms. The first term quasiclassical electrostatic contribution ΔE_{elstat} corresponds to Coulomb interaction the charge density of fragments and the nuclei of the other fragment. The repulsion resulting from antisymmetrization and normalization of the resulting product wave function is called Pauli repulsion ΔE_{Pauli} and the attractive orbital contribution ΔE_{orb} encompass all orbital relaxation effects, such as charge transfer and polarization.

$$\Delta E_{int}(elec) = \Delta E_{elstat} + \Delta E_{Pauli} + \Delta E_{orb} \tag{3}$$

The orbital contributions can be further decomposed using the Natural Orbital for Chemical Valence (NOCV) extension[6]. The resulting NOCV deformation densities reveal the charge flow during bond formation, with the associated energy contributions indicating their significance, and the eigenvalues serving as a measure of the charge transfer. This method helps identify the orbitals involved.

Structures were optimized and subjected to EDA calculation with the Amsterdam Modeling Suite (AMS, version 2021.105).[7] To ensure use of the most optimal conformer for the EDA calculation, a conformer search using CREST[8] was conducted. The energetically most stable conformers were subsequently reoptimized by DFT-based methods and the resulting best conformer chosen for the EDA. All DFT calculations were performed with the B3LYP functional[9] and the all-electron basis set TZP.[10] Additionally, the DFT-D3 dispersion correction with the Becke-Johnson damping function[11] was used. Scalar relativistic effects were treated by the zeroth order regular approximation.[12] The numerical quality was set to “very good” which governs the density fitting and numerical integration. This numerical quality corresponds to $10^{-6} E_\mathrm{h}$ as SCF convergence criterion. For the geometry optimization, this corresponded to the energy criterion of $3 \cdot 10^{-3} E_\mathrm{h}$ and the gradient criterion of $10^{-3} E_\mathrm{h}$ Å^{-1}.

2. Mass spectra

a) NaX, HFIP/HFIP-d_1

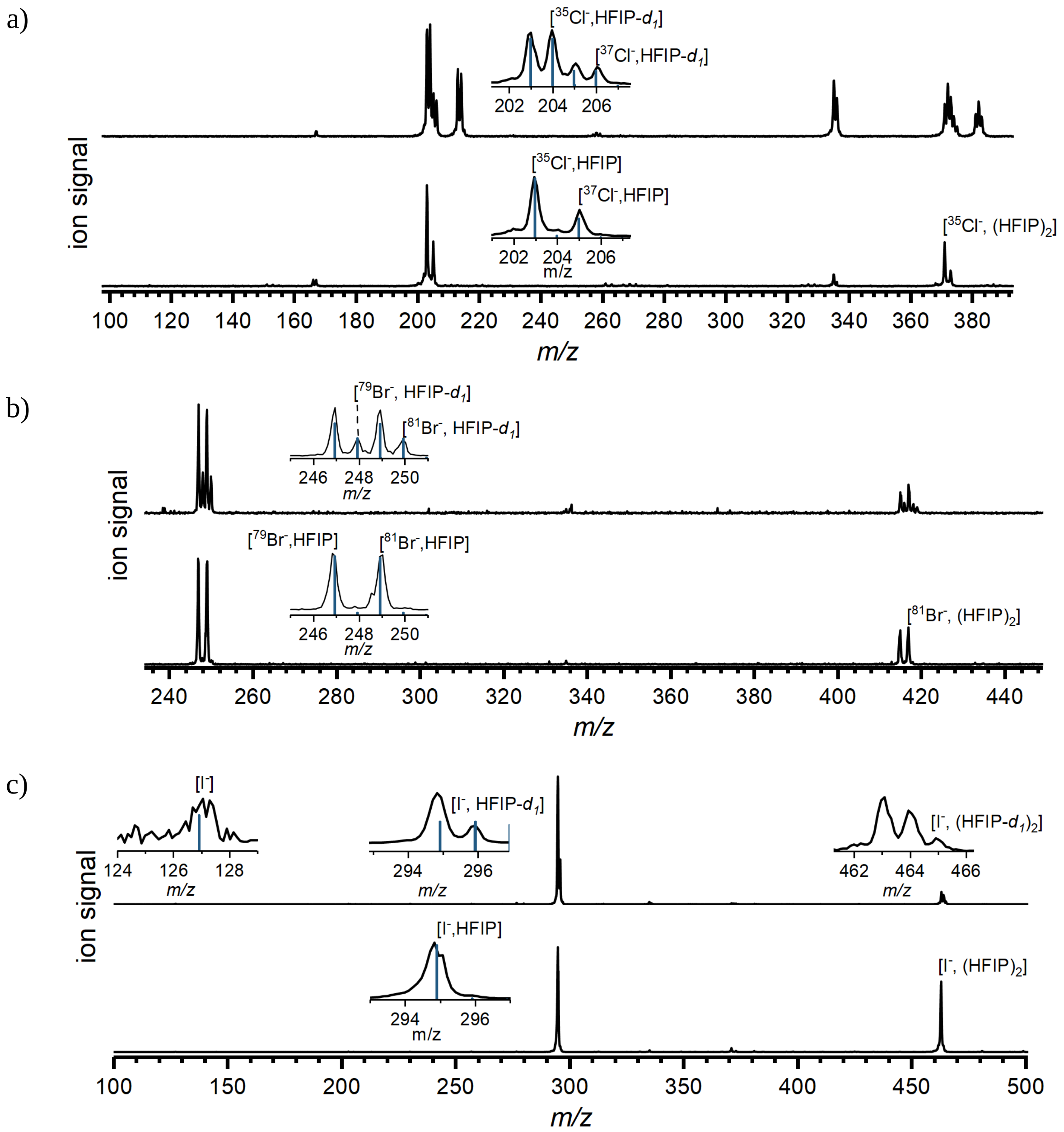

Figure S1 – Quadrupole mass spectra obtained from 0.5 mM NaX and 0.5 mM HFIP solutions in MeOH/H_2O (1:2, v/v) with and without inducing H/D exchange a) NaCl, b) NaBr; c) NaI. Stick spectra (blue) represent theoretical isotopic distribution for selected complexes, based on the halide isotopes natural abundance. Exact mass values were used to calibrate the spectra applying a linear regression with at least 7 points in the range from 30 to 500 *m/z*.

b) NaX, *i*-PrOH/ *i*-PrOD

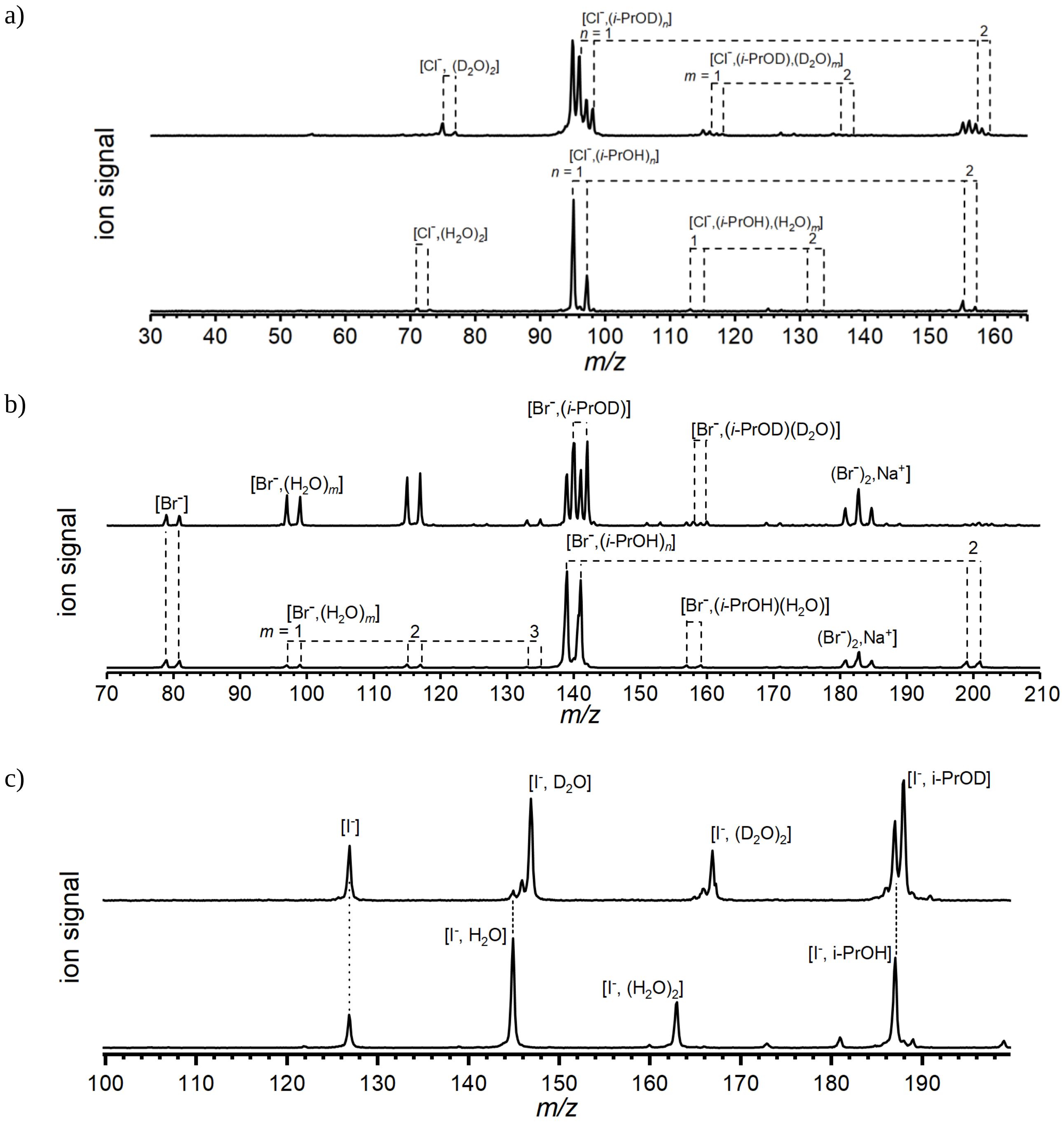

Figure S2 – Quadrupole mass spectra obtained from 0.1 mM NaX solutions in H_2O/*i*-PrOH (1:10, v/v). a) NaCl; b) NaBr; c) NaI. For the spectrum of deuterated species solutions were prepared with same salt concentration in D_2O/ *i*-PrOD and D_2SO_4. Exact mass values were used to calibrate the spectra applying a linear regression with at least 7 points in the range from 30 to 500 *m/z*.

3. Calculated vibrational spectra

3.1 Comparison of X^-(HFIP) isomers: antiperiplanar (AP) *vs* synperiplanar (SP)

a) Cl^-(HFIP) and Cl^-(HFIP-d_1)

i) DVR-FBR, OH/OD stretching vibrational transition spectral region

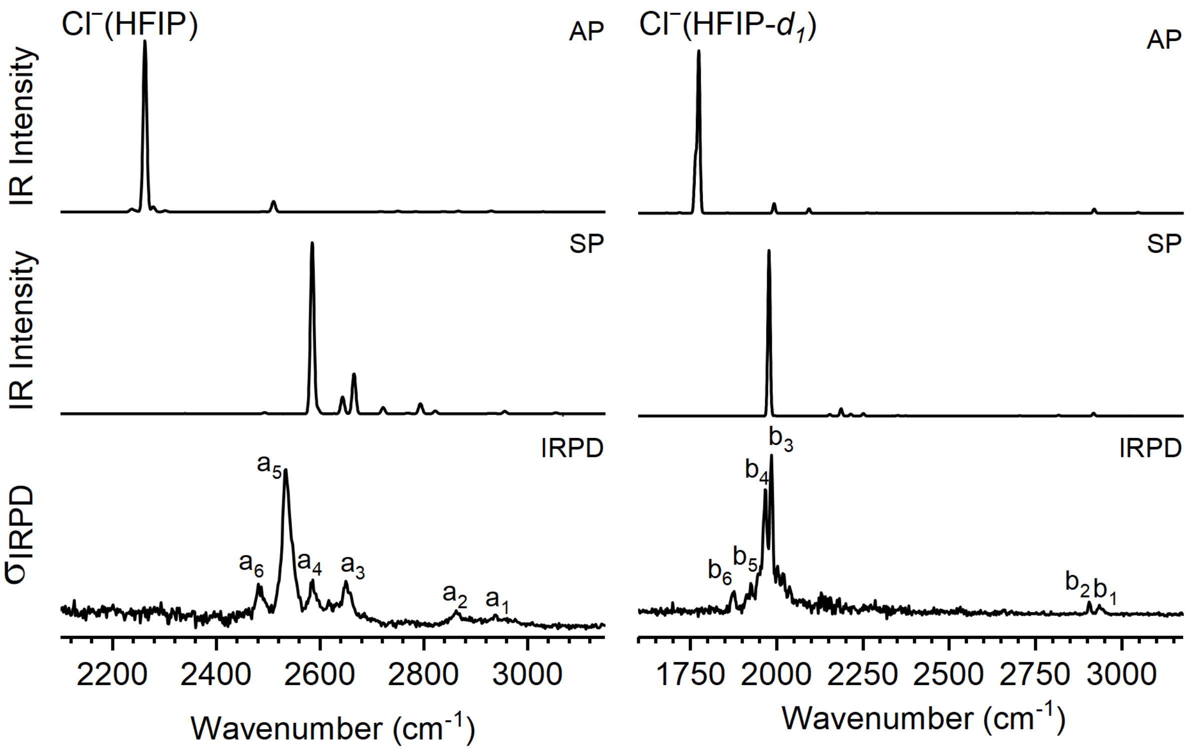

Figure S3 DVR-FBR/RI-MP2+DLPNO-CCSD(T)/aug-cc-pVTZ spectra of the AP (top panel) and the SP isomer (see Figure 2 in the main text for geometries) of Cl^-(HFIP) (left) and Cl^-(HFIP-d_1) (right) compared to the IRPD spectrum of the corresponding D_2-tagged complex. See Table 4 (main text) for band positions, vibrational frequencies and assignments. DVR-FBR spectra were convoluted using a Gaussian line-shape function with a FWHM of 8 cm^{-1}.

b) Br⁻(HFIP) and Br⁻(HFIP-d_1)

i) Harmonic

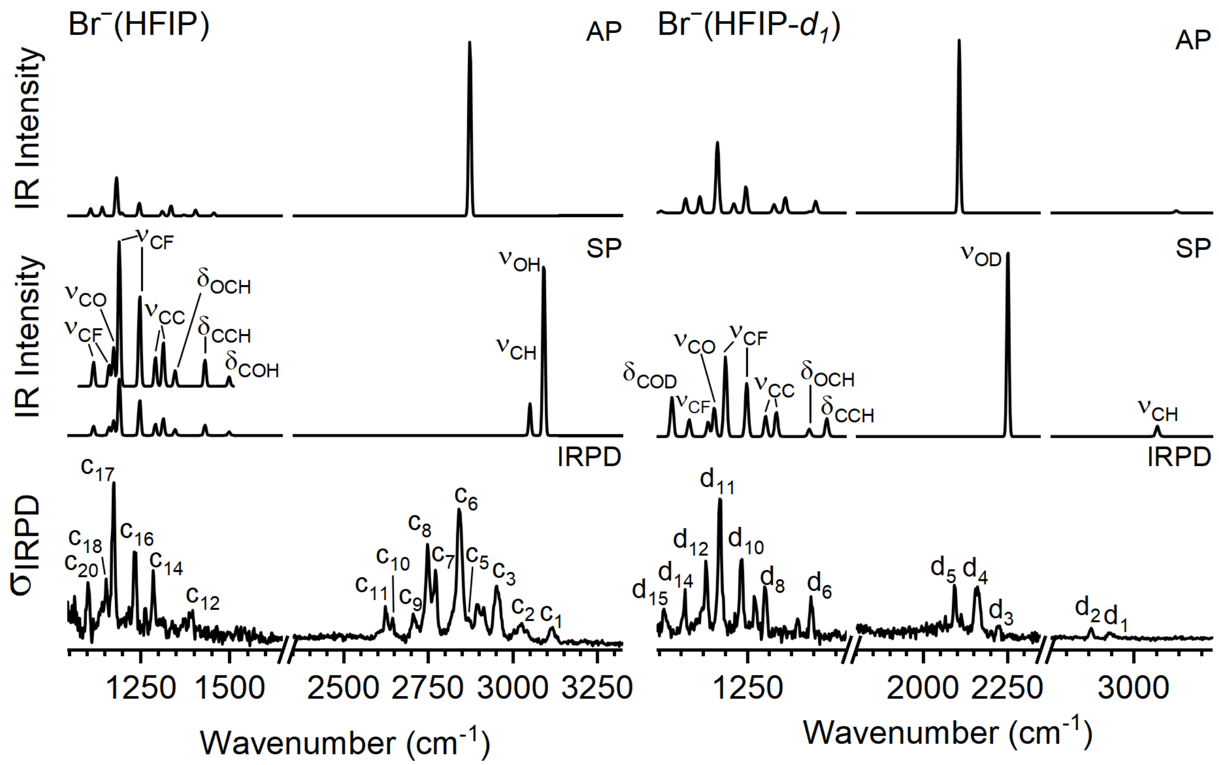

Figure S4 Unscaled harmonic MP2/aug-cc-pVTZ IR spectra of the AP (top panel) and the SP isomer (see Figure 2 for geometries) of Br⁻(HFIP) (left) and Br⁻(HFIP-d_1) (right) compared to the IRPD spectrum of the corresponding D_2-tagged complex. See Tables 1, 3 and 4 (Main text) for band positions, harmonic vibrational frequencies and assignments. The harmonic spectra were convoluted using a Gaussian line-shape function with a FWHM of 8 cm^{-1}.

ii) DVR-FBR, OH/OD stretching vibrational transition spectral region

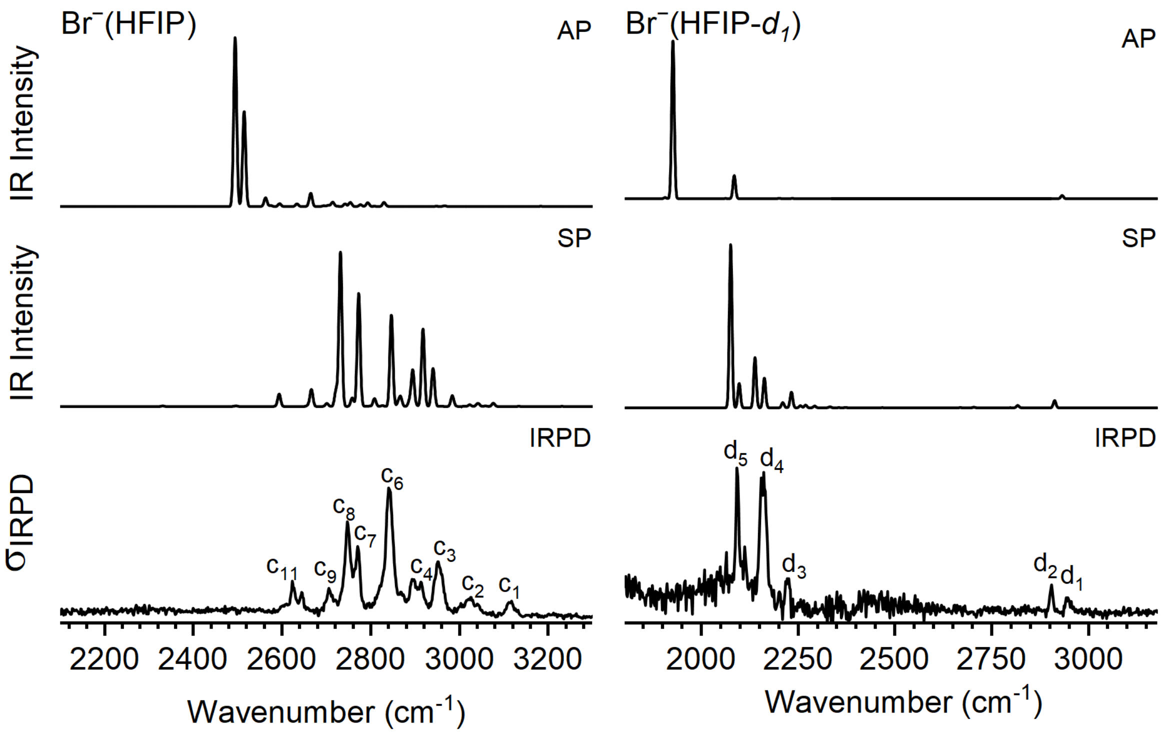

Figure S5 DVR-FBR/RI-MP2+DLPNO-CCSD(T)/aug-cc-pVTZ spectra of the AP (top panel) and the SP isomer (see Figure 2 for geometries) of Br^-(HFIP) (left) and Br^-(HFIP-d_1) (right) compared to the IRPD spectrum of the corresponding D_2-tagged complex in the OH/OD stretching region. See Table 4 (Main text) for band positions, vibrational frequencies and assignments. DVR-FBR spectra were convoluted using a Gaussian line-shape function with a FWHM of 8 cm^{-1}.

c) I$^-$(HFIP) and I$^-$(HFIP-d_1)

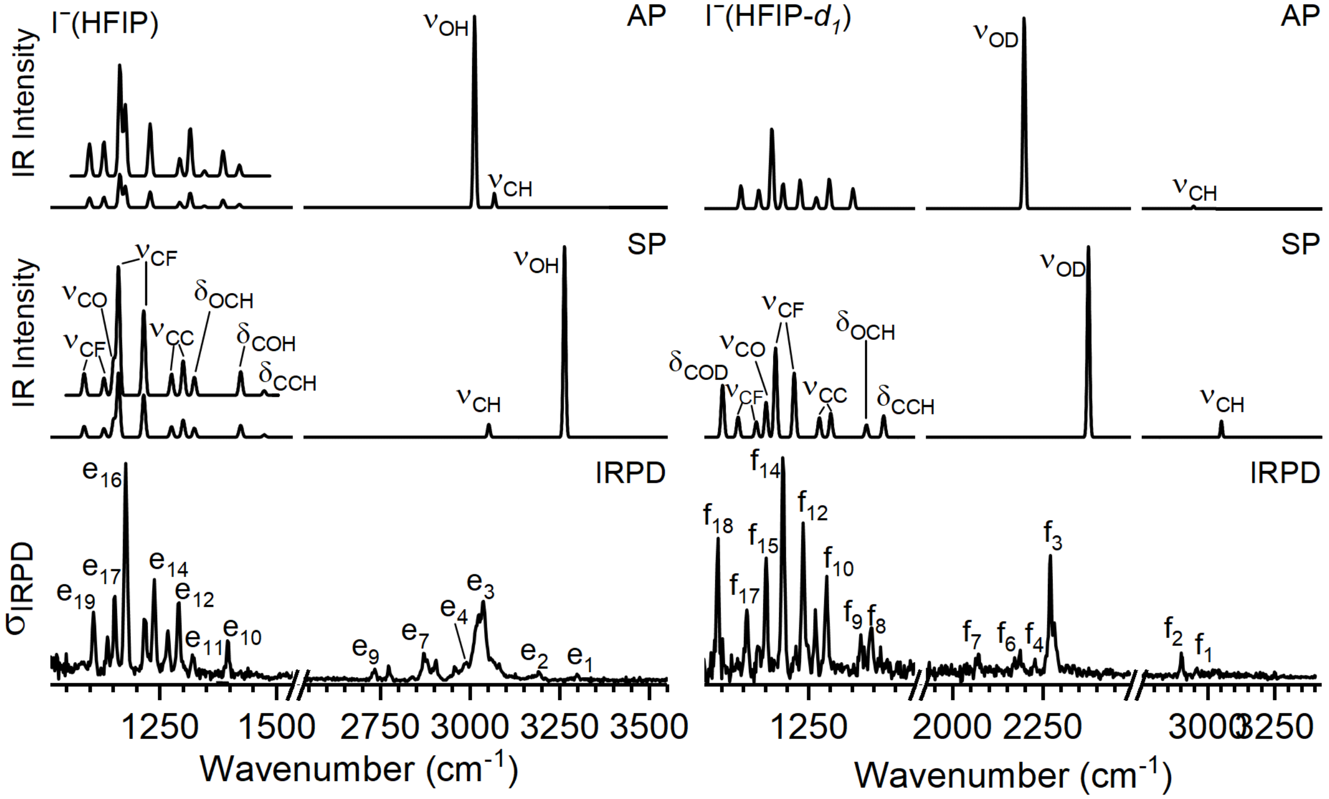

Figure S6 Unscaled harmonic MP2/aug-cc-pVTZ IR spectra of the AP (top panel) and the SP isomer (see Figure 2, main text, for geometries) of I$^-$(HFIP) (left) and I$^-$(HFIP-d_1) (right) compared to the IRPD spectrum of the corresponding D_2-tagged complex. See Table 3 (main text) for band positions, harmonic vibrational frequencies and assignments. The harmonic spectra were convoluted using a Gaussian line-shape function with a FWHM of 8 cm^{-1}.

3.2 X⁻(HFIP), SP: Harmonic *vs.* VPT2 *vs* DVR-FBR predicted spectra

a) Cl⁻(HFIP) and Cl⁻(HFIP-*d₁*)

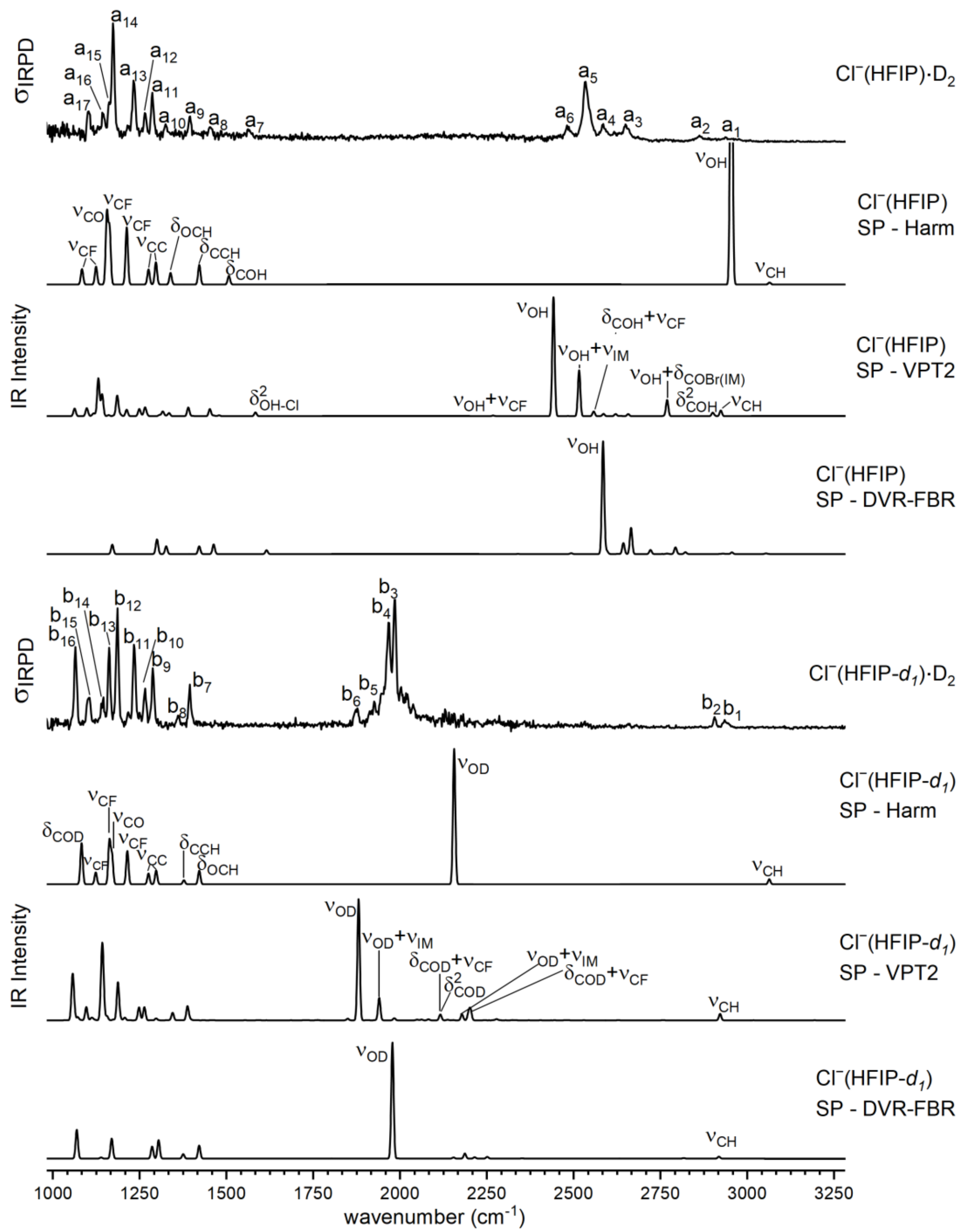

Figure S7 IRPD spectra of D_2-tagged Cl⁻(HFIP) and Cl⁻(HFIP-*d₁*), calculated harmonic MP2/aug-cc-pVDZ, anharmonic VPT2/MP2/aug-cc-pVDZ and anharmonic DVR-FBR/RI-MP2+DLPNO-CCSD(T)/aug-cc-pVTZ spectra of corresponding (unttaged) SP complexes. The simulated spectra were convoluted using a Gaussian line-shape function with a FWHM of 8 cm^{-1}.

b) Br^{-}(HFIP) and Br^{-}(HFIP-d_1)

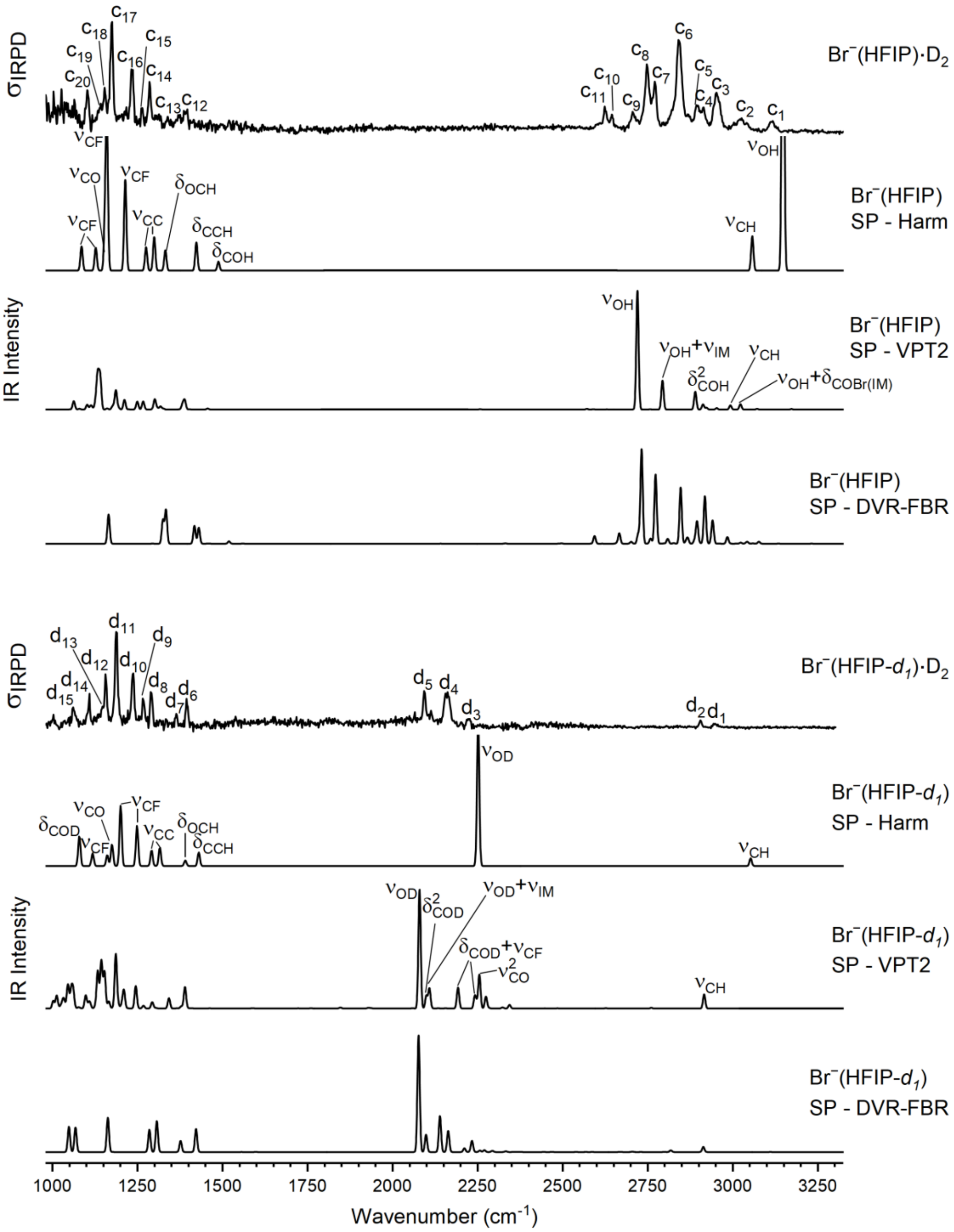

Figure S8 IRPD spectra of D_2-tagged Br^{-}(HFIP) and Br^{-}(HFIP(-d_1) complexes and corresponding harmonic, VPT2/MP2/aug-cc-pVDZ and DVR-FBR/ri-MP2/aug-cc-pVTZ spectra of untagged SP complexes. The simulated spectra were convoluted using a Gaussian line-shape function with a FWHM of 8 cm^{-1}.

c) I⁻(HFIP) and I⁻(HFIP-d_1)

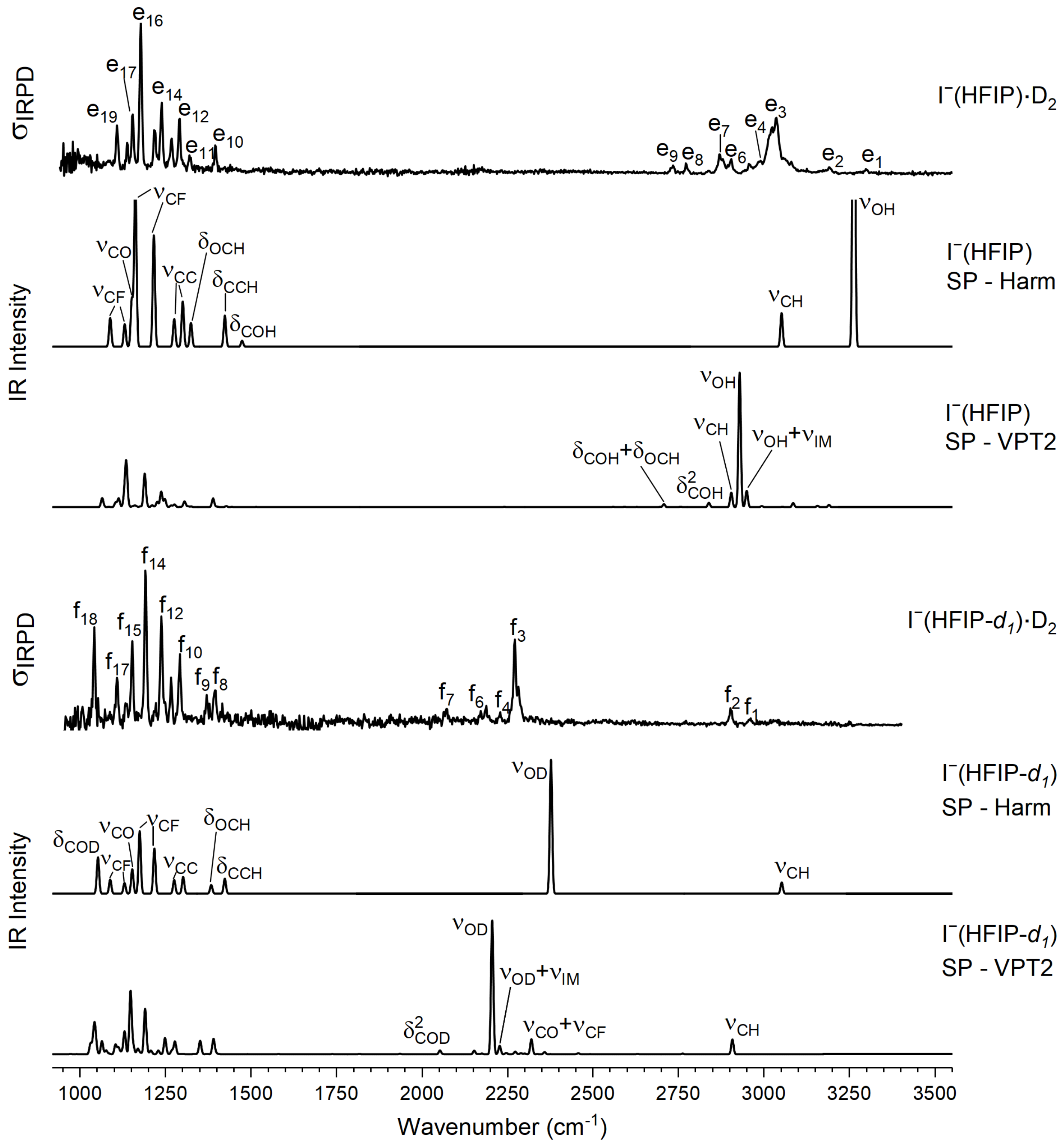

Figure S9 IRPD spectra of D_2-tagged I⁻(HFIP) and I⁻(HFIP-d_1) and calculated harmonic and VPT2/MP2-aug-cc-pVDZ spectra of corresponding untagged SP complexes. The simulated spectra were convoluted using a Gaussian line-shape function with a FWHM of 8 cm^{-1}.

3.3 IPRD vs Harmonic Spectra of X^-(*i*-PrOH) and X^-(*i*-PrOD)

a) Cl^-(*i*-PrOH) and Cl^-(*i*-PrOD)

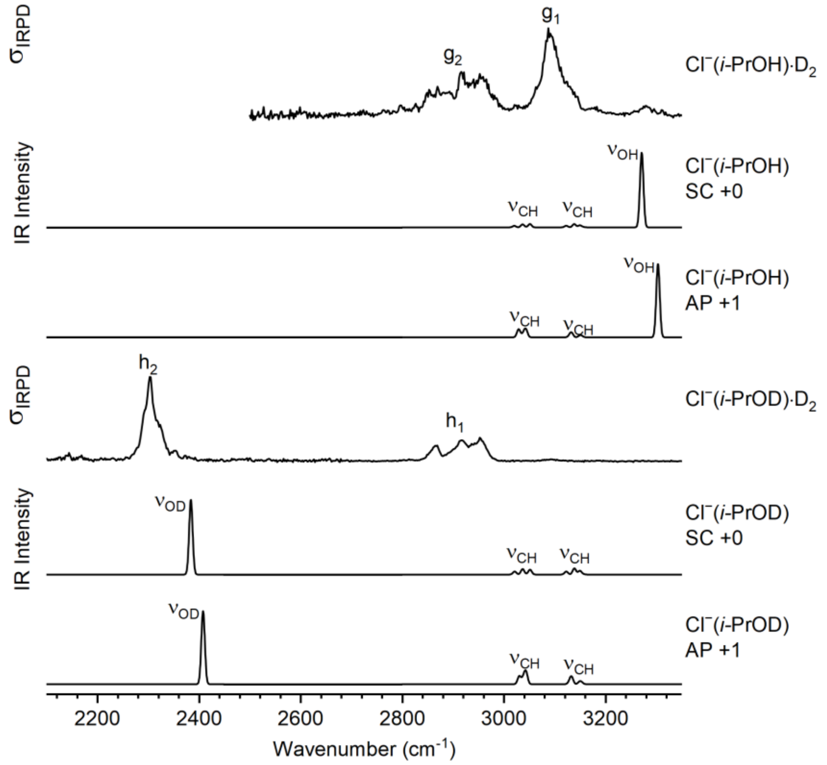

Figure S10 - IRPD Spectra of D_2-tagged Cl^-(*i*-PrOH) and Cl^-(*i*-PrOH(D) compared to unscaled harmonic MP2/aug-cc-pVTZ spectra of corresponding SC and AP untagged complexes, ZPE corrected relative energy of complexes shown in kJ mol^{-1}. The simulated spectra were convoluted using a Gaussian line-shape function with a FWHM of 8 cm^{-1}.

Table S1 Band labels, IRPD band positions, harmonic MP2/aug-cc-pVTZ vibrational frequencies (in cm^{-1}) and band assignments of the fundamental transitions in the CH/OH(D) stretching region. Values for the corresponding deuterated isotopologue are given in parentheses. If no value is given in parenthesis assume values are identical to the value obtained for the H-isotopologue.

Label	Band Position	Harm. Freq.	Assignment
g_2 (h_1)	2832 – 2997<br>(2985 - 2839)	3020(3021), 3036, 3051, 3122, 3138, 3149, 3156	$7x\ \nu_{CH}$, $1x\ \nu_{DD}$
g_1 (h_2)	3091 (2302)	3271 (2383)	ν_{OH} (ν_{OD})

b) Br^-(*i*-PrOH) and Br^-(*i*-PrOD)

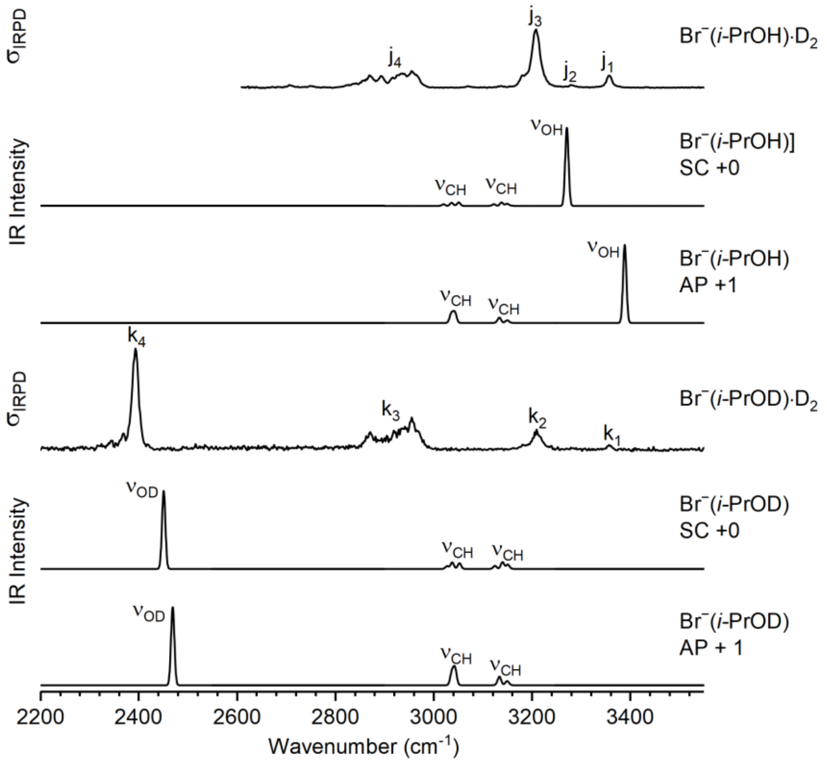

Figure S11 - IRPD spectra of D_2-tagged Br^-(*i*-PrOH) and Br^-(*i*-PrOD) compared to harmonic MP2/aug-cc-pVTZ spectra of corresponding SC and AP untagged complexes. ZPE corrected relative energy of complexes shown in kJ mol^{-1}. The simulated spectra were convoluted using a Gaussian line-shape function with a FWHM of 8 cm^{-1}.

Table S2 - Band labels, IRPD band positions, harmonic MP2/aug-cc-pVTZ vibrational frequencies (in cm^{-1}) and band assignments of the fundamental transitions in the CH/OH(D) stretching region. Values for the corresponding deuterated isotopologue are given in parentheses. If no value is given in parenthesis it is identical to the value obtained for the H-isotopologue.

Label	Band Position	Harm. Freq.	Assignment
j_4 (k_3)	2810-2988 (2852-2991)	3027, 3038, 3052, 3124, 3140, 3150, 3154	$7x\ \nu_{CH}$, ν_{DD}
j_3 (k_4)	3207 (2393)	3365 (2450)	ν_{OH} (ν_{OD})

c) I⁻ (*i*-PrOH) and I⁻ (*i*-PrOD)

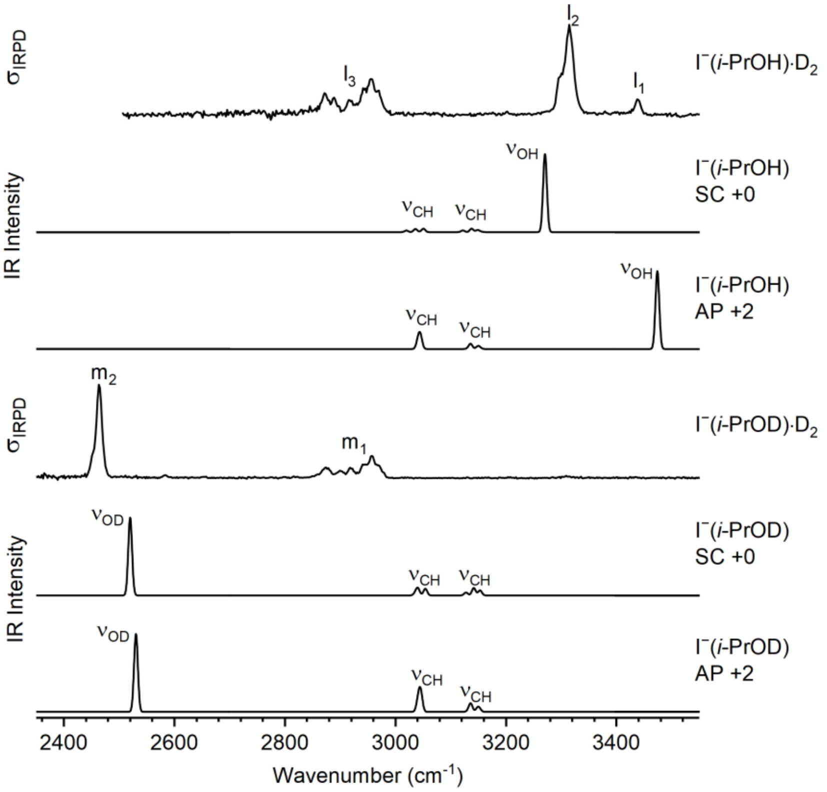

Figure S12 - IRPD Spectra of D_2-tagged I^-(*i*-PrOH) and I^-(*i*-PrOD) compared to harmonic /MP2-aug-cc-pVTZ spectra of corresponding untagged SC and AP complexes. ZPE corrected relative energy of complexes shown in kJ mol^{-1}. The simulated spectra were convoluted using a Gaussian line-shape function with a FWHM of 8 cm^{-1}.

Table S3 Band labels, IRPD band positions, harmonic MP2/aug-cc-pVTZ vibrational frequencies (in cm^{-1}) and band assignments of the fundamental transitions in the CH/OH(D) stretching region. Values for the corresponding deuterated isotopologue are given in parentheses.

Label	Band Position	Harm. Freq.	Assignment
l_3, (m_1)	2845 – 2993<br>(2843 – 2988)	3036, 3041, 3054, 3128, 3142, 3153, 3154	$7x\ \nu_{CH}$, $1x\ \nu_{DD}$
l_2 (m_2)	3315 (2463)	3460 (2520)	ν_{OH} (ν_{OD})

4. Tag Effect

a) IRPD Br^{-}(HFIP) - H_2 *vs* D_2 tag

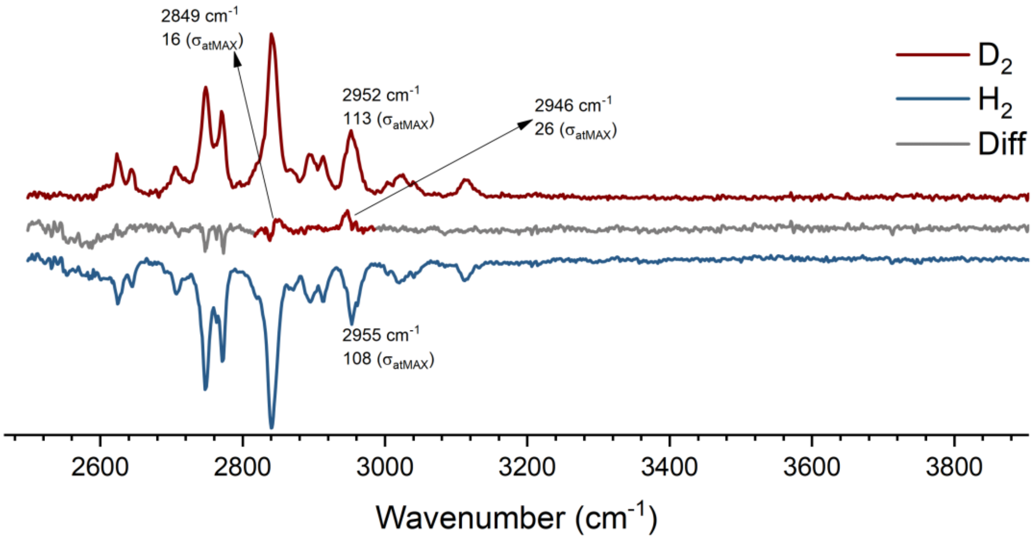

Figure S13: IRPD spectra of D_2- (top, red) and H_2- (bottom, blue) tagged Br^{-}(HFIP) complexes, and difference spectrum (middle, gray). Highlighted in red are two possible positions of the DD stretch.

b) Calculated tag effect

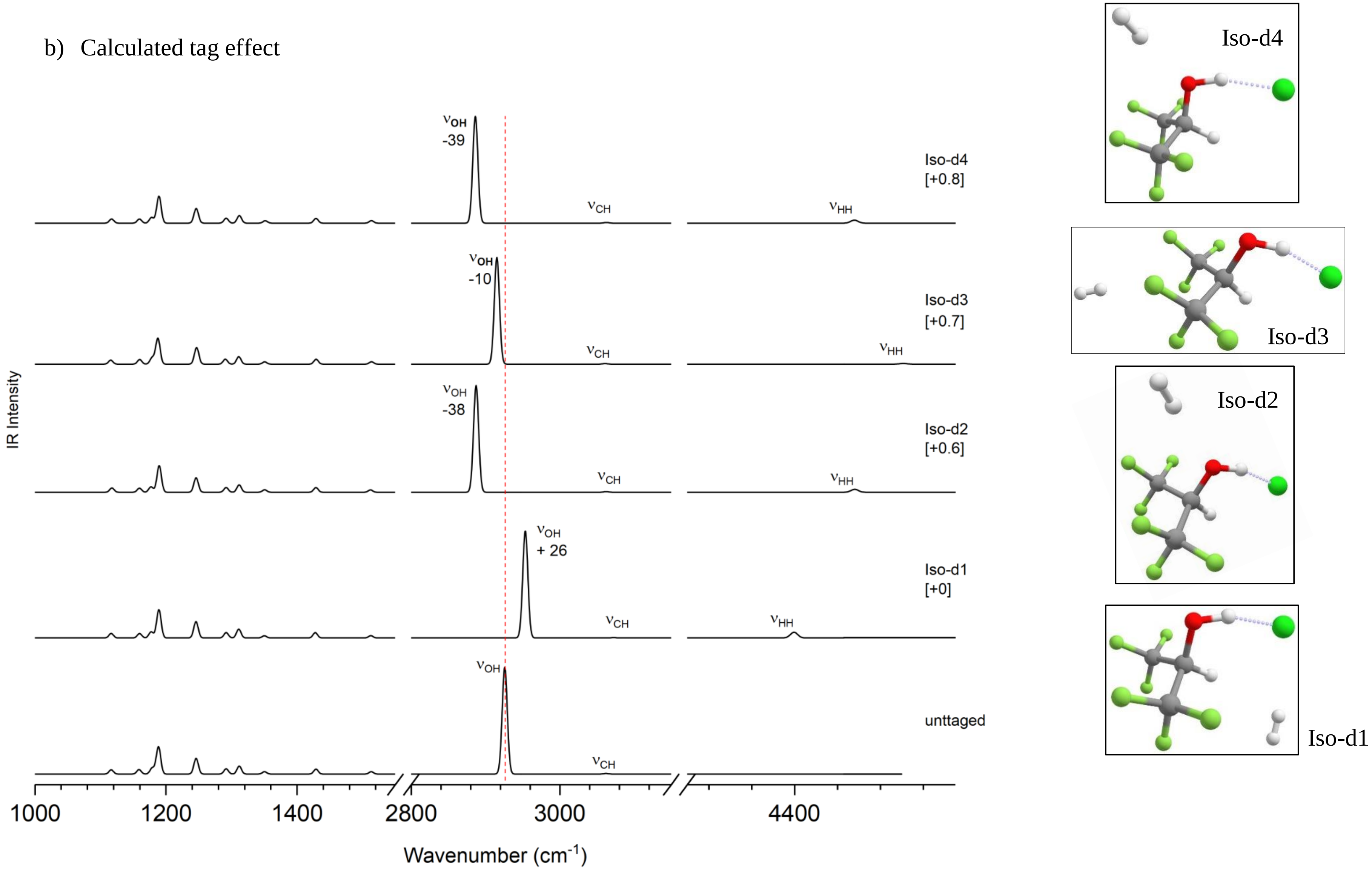

Figure S14: MP2/aug-cc-pVTZ harmonic IR spectra of untagged and H_2-tagged Cl^-(HFIP) low-energy isomers (relative energy in brackets in kJ mol^{-1}) Tag-induced frequency shift (in cm^{-1}) of the OH stretching transition indicated.

5. Comparison between EDA results and BSSE corrected MP2 dissociation energies

Table S4 Comparison of "dissociation energies" obtained using EDA (E_{bond}) or MP2/aTZ complexation energy after Counterpoise Correction

	X^{-}-H_2O			X^{-}-HFIP			X^{-}-*i*-PrOH		
X^{-}	Cl^{-}	Br^{-}	I^{-}	Cl^{-}	Br^{-}	I^{-}	Cl^{-}	Br^{-}	I^{-}
ΔE_{prep}	+3	+2	+2	+23	+19	+16	+1	+3	+3
Deformation.	+2	+2	+1	+20	+17	+15	+4	+3	+3
E_{bond}	-71	-60	-51	-147	-123	-101	-90	-67	-54
$\Delta E_{Complex(CP)}$	-54	-46	-38	-124	-110	-94	-69	-52	-49
d(X^{-} -H) B3LYP	213	233	264	192	214	242	215	235	261
d(X^{-} -H) MP2/a-TZ	212	229	256	192	211	235	207	234	250

[a] Energies in kJ mol^{-1} and bond length in pm.

6. 5. Energy Decomposition Analysis

a) Br^-(HM)

Table S5 EDA results of hydrogen bonds between H_2O, HFIP, *i*-PrOH and the Br^- anion

	Bromide					
	H_2O		HFIP		*i*-PrOH	
ΔE_{int}	-62		-142		-70	
$\Delta E_{int}(disp)$[b]	-4	(6%)	-10	(7%)	-11	(16%)
$\Delta E_{int}(elec)$[b]	-58	(94%)	-132	(93%)	-59	(84%)
ΔE_{Pauli}	+46		+96		+67	
ΔE_{elstat}[c]	-69	(67%)	-149	(65%)	-75	(59%)
ΔE_{orb}[c]	-34	(33%)	-79	(35%)	-52	(41%)
$\Delta E_1(Br^- \rightarrow H\text{-}O)$ [d]	-24	(73%)	-49	(62%)	-26	(53%)
$\Delta E_2(Br^- \rightarrow H\text{-}C)$ [d]			-7	(9%)	-6	(12%)
$\Delta E_3(Br^- \rightarrow H\text{-}C$[d])					-4	(8%)
ΔE_{prep}	+2		+19		+3	
E_{bond}	-60		-123		-67	
$d(Br^- - H)$	2.33		2.14		2.35	

[a] Energies in kJ mol-1 and bond length in Å.

[b] Percentage values give the relative contributions of dispersion and electronic effects to ΔE_{int}.

[c] Percentage values give the relative contributions to the attractive EDA terms ΔE_{elstat} and ΔE_{orb}.

[d] Percentage values give the relative contributions of the NOCV to ΔE_{orb}

b) I^-(HM)

Table S6 EDA results of hydrogen bonds between H_2O, HFIP, *i*-PrOH and the I^- anion

	Iodide					
	H_2O		HFIP		*i*-PrOH	
ΔE_{int}	-49		-117		-57	
ΔE_{int}(disp)[b]	-5	(10%)	-12	(10%)	-11	(19%)
ΔE_{int}(elec)[b]	-44	(90%)	-105	(90%)	-46	(81%)
ΔE_{Pauli}	+34		+82		+50	
ΔE_{elstat}[c]	-56	(71%)	-125	(67%)	-61	(64%)
ΔE_{orb}[c]	-23	(29%)	-62	(33%)	-35	(36%)
$\Delta E_1(I^- \rightarrow H\text{-}O)$ [d]	-17	(74%)	-41	(66%)	-21	(60%)
$\Delta E_2(I^- \rightarrow H\text{-}C)$ [d]			-6	(10%)	-3	(9%)
$\Delta E_3(I^- \rightarrow H\text{-}C$[d])					-3	(9%)
ΔE_{prep}	+2		+16		+3	
E_{bond}	-51		-101		-54	
$d(\mathrm{Br}^- - \mathrm{H})$	2.64		2.42		2.61	

[a] Energies in kJ mol-1 and bond length in Å.

[b] Percentage values give the relative contributions of dispersion and electronic effects to ΔE_{int}.

[c] Percentage values give the relative contributions to the attractive EDA terms ΔE_{elstat} and ΔE_{orb}.

[d] Percentage values give the relative contributions of the NOCV to ΔE_{orb}

All NOCVs have a similar shape to those in the main paper for Chloride (Figure 6).

7. Anion Proton Affinity

$$-\Delta PA = PA(M^-) - PA(X^-) \qquad \text{Eq 1}$$

$$X^-(HM) \rightarrow HX + M^- \qquad (1)$$

Table S7 – Difference from experimental anion proton affinities as defined in Eq 1 from ref[13] with values from ref[14] and ZPE corrected MP2/aug-cc-pVTZ energy difference (enthalpy) for reaction (1)

**X^-/ Solvent (HM)**	**-ΔPA**			**$\Delta_r H_{(ZPE)}$**			**$\Delta_r H_{(ZPE)}$**		
	H_2O	HFIP	*i*-PrOH	H_2O	HFIP	*i*-PrOH	D_2O	HFIP-d_1	*i*-PrOD
**Cl^-**	227	48	174	227	47	176	231	50	179
**Br^-**	269	90	216	262	81	211	266	85	214
**I^-**	307	128	254	300	119	249	305	124	253

[a] reaction enthalpy calculated from zero-point energy corrected electronic energies, values presented in kJ mol^{-1}

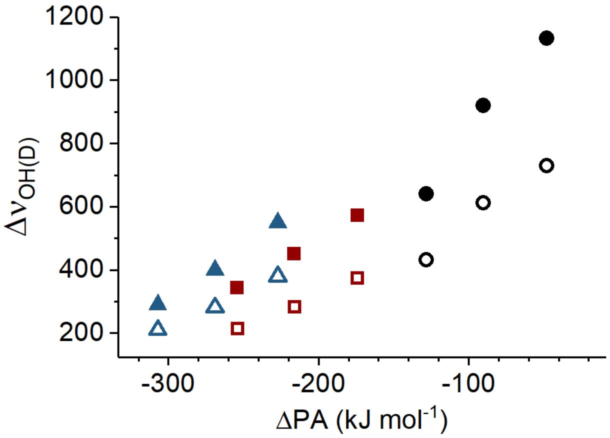

Figure S15 Shift relative to free OH ($\Delta\nu_{OH}$, solid) or free OD ($\Delta\nu_{OD}$, hollow) *vs* ΔPA for X^-(HM) complexes, X^- = Cl^-, Br^-, I^-, HM = HFIP (circles, black), isopropanol (squares, red), and water (triangles, blue).

8. References

1 S. Carter, J. M. Bowman and N. C. Handy, *Theor. Chem. Acc.*, 1998, **100**, 191.

2 F. Neese, *WIREs. Comput. Mol. Sci.*, 2012, **2**, 73.

3 J. C. Light and T. Carrington, in *Advances in Chemical Physics*, ed. I. Prigogine and S. A. Rice, Wiley, 2000, vol. 114, pp. 263–310.

4 Q.-R. Huang, K. Yano, Y. Yang, A. Fujii and J.-L. Kuo, *Phys. Chem. Chem. Phys.*, 2024, **26**, 10757.

5 P. Virtanen, R. Gommers, T. E. Oliphant, M. Haberland, T. Reddy, D. Cournapeau, E. Burovski, P. Peterson, W. Weckesser, J. Bright, S. J. van der Walt, M. Brett, J. Wilson, K. J. Millman, N. Mayorov, A. R. J. Nelson, E. Jones, R. Kern, E. Larson, C. J. Carey, İ. Polat, Y. Feng, E. W. Moore, J. VanderPlas, D. Laxalde, J. Perktold, R. Cimrman, I. Henriksen, E. A. Quintero, C. R. Harris, A. M. Archibald, A. H. Ribeiro, F. Pedregosa and P. van Mulbregt, *Nature methods*, 2020, **17**, 261.

6 M. P. Mitoraj, A. Michalak and T. Ziegler, *J. Chem. Theory Comput.*, 2009, **5**, 962.

7 G. te Velde, F. M. Bickelhaupt, E. J. Baerends, C. Fonseca Guerra, S. J. A. van Gisbergen, J. G. Snijders and T. Ziegler, *J. Comput. Chem.*, 2001, **22**, 931.

8 P. Pracht, F. Bohle and S. Grimme, *Phys. Chem. Chem. Phys.*, 2020, **22**, 7169.

9 a) A. D. Becke, *J. Chem. Phys*, 1993, **98**, 5648; b) P. J. Stephens, F. J. Devlin, C. F. Chabalowski and M. J. Frisch, *J. Phys. Chem.*, 1994, **98**, 11623;

10 E. van Lenthe and E. J. Baerends, *J. Comput. Chem.*, 2003, **24**, 1142.

11 a) S. Grimme, J. Antony, S. Ehrlich and H. Krieg, *J. Chem. Phys*, 2010, **132**; b) S. Grimme, S. Ehrlich and L. Goerigk, *J. Comput. Chem.*, 2011, **32**, 1456;

12 E. van Lenthe, A. Ehlers and E.-J. Baerends, *J. Chem. Phys*, 1999, **110**, 8943.

13 in *The IUPAC Compendium of Chemical Terminology*, ed. V. Gold, International Union of Pure and Applied Chemistry (IUPAC), Research Triangle Park, NC, 2019.

14 P.J. Linstrom and W.G. Mallard, ed., *"Proton Affinity Evaluation" in NIST Chemistry WebBook, NIST Standard Reference Database Number 69*, Gaithersburg MD.
